# Sesquiterpenes and Sesquiterpene Derivatives from *Ferula*: Their Chemical Structures, Biosynthetic Pathways, and Biological Properties

**DOI:** 10.3390/antiox13010007

**Published:** 2023-12-19

**Authors:** Junchi Wang, Qi Zheng, Huaxiang Wang, Leiling Shi, Guoping Wang, Yaqin Zhao, Congzhao Fan, Jianyong Si

**Affiliations:** 1 The Key Laboratory of Bioactive Substances and Resources Utilization of Chinese Herbal Medicine, Ministry of Education, Institute of Medicinal Plant Development, Chinese Academy of Medical Sciences & Peking Union Medical College, Beijing 100193, China; jcwang@implad.ac.cn (J.W.); zhengqi@implad.ac.cn (Q.Z.); wanghuaxiang@implad.ac.cn (H.W.); 2 Xinjiang Institute of Chinese Materia Medica and Ethnodrug, Urumqi 830002, China; shileiling@sina.com (L.S.); ping112_003@163.com (G.W.); xjzyq123@126.com (Y.Z.)

**Keywords:** *Ferula*, sesquiterpene, biosynthetic pathway, antioxidative, antibacterial, anti-inflammatory

## Abstract

*Ferula* is a genus of flowering plants known for its edible and medicinal properties. Since ancient times, many species of *Ferula* have been used in traditional medicine to treat various health issues across countries, such as digestive disorders, respiratory problems, and even as a remedy for headaches and toothaches. In addition, they are also used as a flavoring agent in various cuisines. As the main active ingredients in *Ferula*, sesquiterpenes and their derivatives, especially sesquiterpene coumarins, sesquiterpene phenylpropanoids, and sesquiterpene chromones, have attracted the attention of scientists due to the diversity of their chemical structures, as well as their extensive and promising biological properties, such as antioxidative, anti-inflammatory, antibacterial properties. However, there has not been a comprehensive review of sesquiterpenes and their derivatives from this plant. This review aims to provide an overview of the chemical structures, biosynthetic pathways, and biological properties of sesquiterpenes and sesquiterpene derivatives from *Ferula*, which may help guide future research directions and possible application methods for this valuable edible and medicinal plant.

## 1. Introduction

*Ferula* is a diverse genus of flowering plants belonging to the Apiaceae family, which primarily grow in dry and temperate regions of the Euro-Asian continent, surrounded by India and China in the east, the Canary Islands in the west, Central Europe in the north, and North Africa in the south [1]. This genus comprises about 180 recognized species [2], and it is renowned for its distinctive and often aromatic plants due to the presence of volatile essential oils and sulfide compounds. There are 94 species distributed in the erstwhile USSR, 32 species in Iran, 25 species in China, 19 species in the western Himalayas, 18 species in Turkey, 15 species in Pakistan, 4 species in Saudi Arabia, and 3 species in India [3]. Among these, 15 species are endemic to Iran, 9 species to Turkey, 7 species to China, and 1 species to Italy [4].

Since ancient times, different species of *Ferula* have been used in traditional medicine to treat various diseases across countries [5]. Asafoetida is an oleo–gum–resin obtained from the stems of *Ferula* plants, and in many parts of the world, it is used as a traditional medicine and as a flavoring agent in various cuisines [6]; its dual role in cuisine and traditional medicine is notable in several cultures. Asafoetida is commonly used in Indian, Iranian, and some Middle Eastern dishes, and it is known for enhancing the flavor of dishes, especially in vegetarian recipes. It is a common ingredient in spice blends and seasoning for lentils, vegetables, and rice dishes [7,8]. In the folk medicine of Russia, Iran, China, Turkey, Pakistan, and India, asafoetida is often called “Asafetida”, “Rechina fena (Zaz)”, “A-wei”, “Setan bokosu (Seytan tersi)”, “Anjadana (Kama, Anguza)”, and “Hengu (Hing, Hingu, Ingu, etc.)”, respectively [9]. It is traditionally used to treat various health issues, such as digestive disorders, respiratory problems, and even as a remedy for headaches and toothaches [10,11,12]. The digestive-stimulating effect of Asafoetida is the most common beneficial physiological effect. In addition, other parts of some *Ferula* species also have edible and medicinal values. For instance, some nomadic peoples in central Iran use fried aerial parts of *F. assafoetida* and some seasonings as carminative foods. In Brazil, a hot-water extract from the dried stems and leaves of *F. assafoetida* is used as an aphrodisiac that is orally taken for the treatment of erectile dysfunction [13]. People in Pakistan extensively use the *F. narthex* Boiss herb for the treatment of coughs, fever, scorpion stings, hysteria, gastric dysfunction, constipation, habitual miscarriage, and toothache [14]. In Saudi Arabia, the rhizomes of *F. communis* are called alkalakh, which are used locally as a traditional medicine to treat skin infections, while its roasted flower buds are used to treat fever and dysentery [15]. In Lebanon and Syria, the roots of *F. hermonis* Boiss are used in folk medicine to reduce plasma cholesterol levels and total weight, as well as to treat skin infections, stomach diseases, erectile dysfunction, fever, dysentery, frostbite, and hysteria [16].

Phytochemical studies of the oleo–gum–resin, roots, seeds, and aerial parts of more than 70 species have revealed coumarins [17], phenylpropanoids [18], lignans [19], steroidal esters [20], organic acid glycosides [20], aromatic acids [21], sesquiterpenes [22], monoterpenes [23], benzofurans [24], and sulfur-containing derivatives [25] of the *Ferula* genus. Among these constituents, sesquiterpenes and their derivatives, especially sesquiterpene coumarins, sesquiterpene phenylpropanoids, and sesquiterpene chromones, have attracted the attention of scientists due to the diversity of their chemical structures, as well as their extensive and promising biological properties, such as antioxidative [26], anti-inflammatory [27], antibacterial [28], antitumor [29], and antiviral [30] properties.

While they are the main active ingredients in *Ferula*, there has not, however, been a comprehensive review of sesquiterpenes and their derivatives from this plant. In this review, we aim to report the chemical structures, biosynthetic pathways, and biological properties of sesquiterpenes and sesquiterpene derivatives from *Ferula*. Overall, the purpose of this work is to provide a comprehensive introduction to the bioactive sesquiterpenes of *Ferula*, which may help guide future research directions and possible application methods for this valuable edible and medicinal plant.

## 2. Chemical Structures

### 2.1. Sesquiterpenes

**Figure 1:**
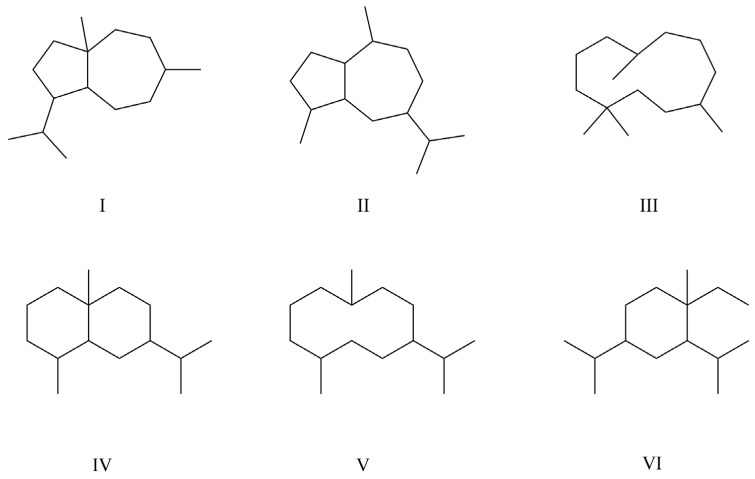
The main structural types of sesquiterpenes in *Ferula* plants (the daucane-type (**I**), guaiane-type (**II**), humulane-type (**III**), eudesmane-type (**IV**), germacrane-type (**V**), and elemane-type (**VI**) sesquiterpenes).

*Ferula* species are known for their production of various secondary metabolites, including sesquiterpenes. Sesquiterpenes are a class of terpenes composed of three isoprene units. The structural types of sesquiterpenes in *Ferula* are dominated by monocyclic and bicyclic sesquiterpenes, such as the daucane-type (I), guaiane-type (II), humulane-type (III), eudesmane-type (IV), germacrane-type (V), and elemane-type (VI) sesquiterpenes (Figure 1). Among them, the daucane-type sesquiterpene is the most common skeleton type.

**Figure 2:**
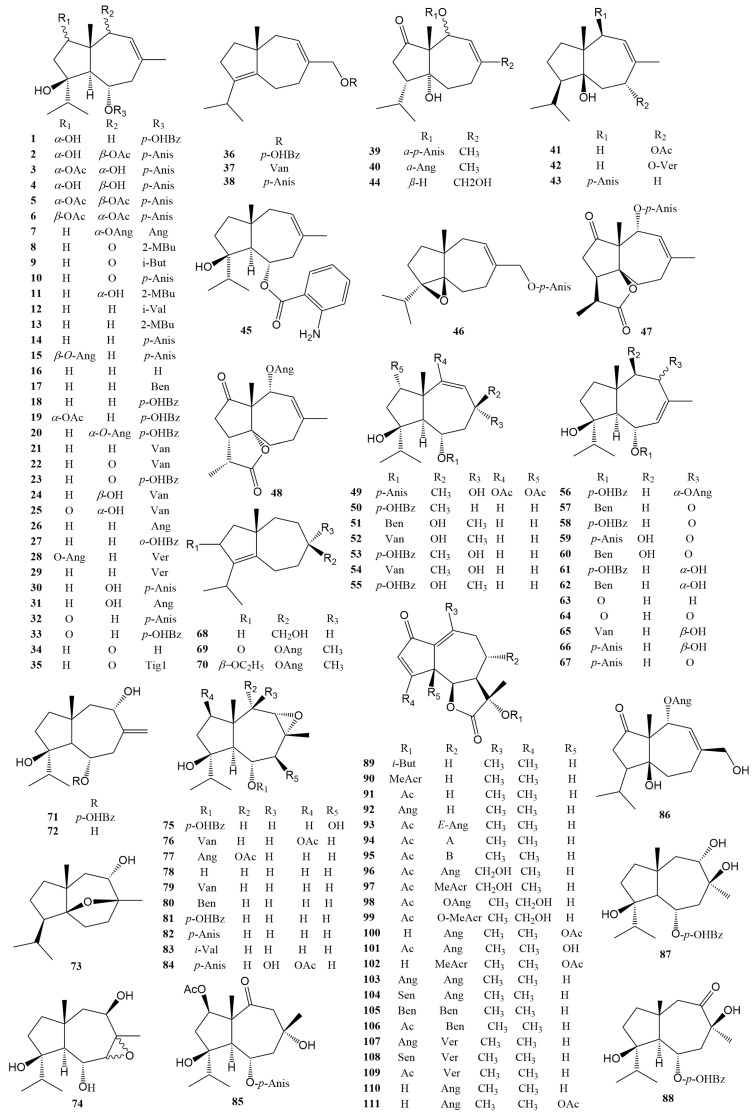

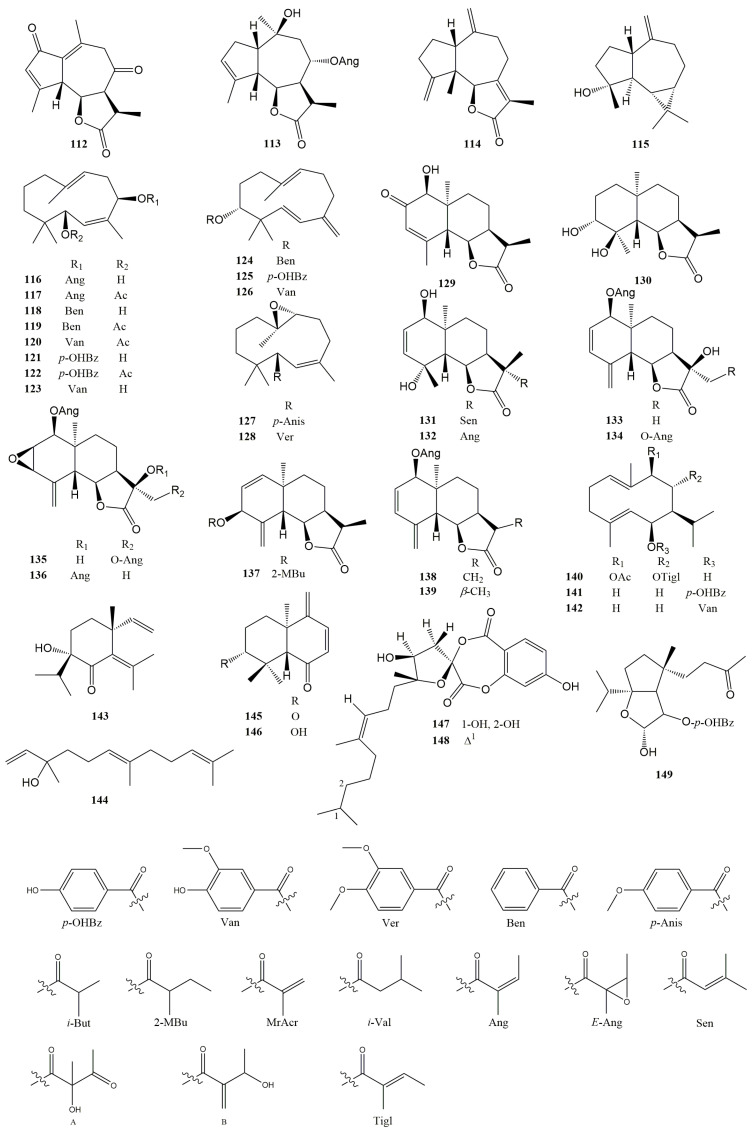
Sesquiterpenes in *Ferula* plants.

Except for a few compounds, the sesquiterpenes in *Ferula* mostly exist in the form of esters, with substituents including fatty acids, aromatic acids, etc. Due to the presence of multiple substituent sites and substituents, the structures of sesquiterpenes are diverse. Here, sesquiterpenes with medicinal or potential medicinal prospects are summarized, including 88 daucane-type sesquiterpenes (**1**–**88**), 27 guaiane-type sesquiterpenes (**89**–**115**), 13 humulane-type sesquiterpenes (**116**–**128**), 11 eudesmane-type sesquiterpenes (**129**–**139**), 3 germacrane-type sesquiterpenes (**140**–**142**), 1 elemane-type sesquiterpene (**143**), and 6 other types of sesquiterpenes (**144**–**149**). The names and sources of compounds **1**–**149** are listed in Table 1, and their chemical structures are shown in Figure 2.

### 2.2. Sesquiterpene Coumarins

Sesquiterpene coumarins are often found in *Ferula* plants and are known for their unique chemical structures and potential bioactivity. According to the connection site between the sesquiterpene unit and the coumarin skeleton, sesquiterpene coumarins can be classified into those connected by a 7-position C-O-C bridge (I) (**150**–**345**), those connected by a 4-position C-O-C bridge (II) (**346**–**348**), and those connected by a 3-position C-C bond (III) (**349**–**407**). According to the structural types of sesquiterpenes, type I compounds can be further classified into a straight-chain type (Ia) (**150**–**175**), monocyclic type (Ib) (**176**–**219**), and bicyclic type (Ic) (**220**–**345**). Type III compounds can be classified into straight-chain coumarin type (IIIa) (**349**–**361**), furanocoumarin type (IIIb) (**362**–**399**), and pyranocoumarin type (IIIc) (**400**–**407**) compounds depending on whether the hydroxyl group in the sesquiterpene moiety forms a five- or six-membered heterocyclic ring with the coumarin moiety.

**Figure 3:**
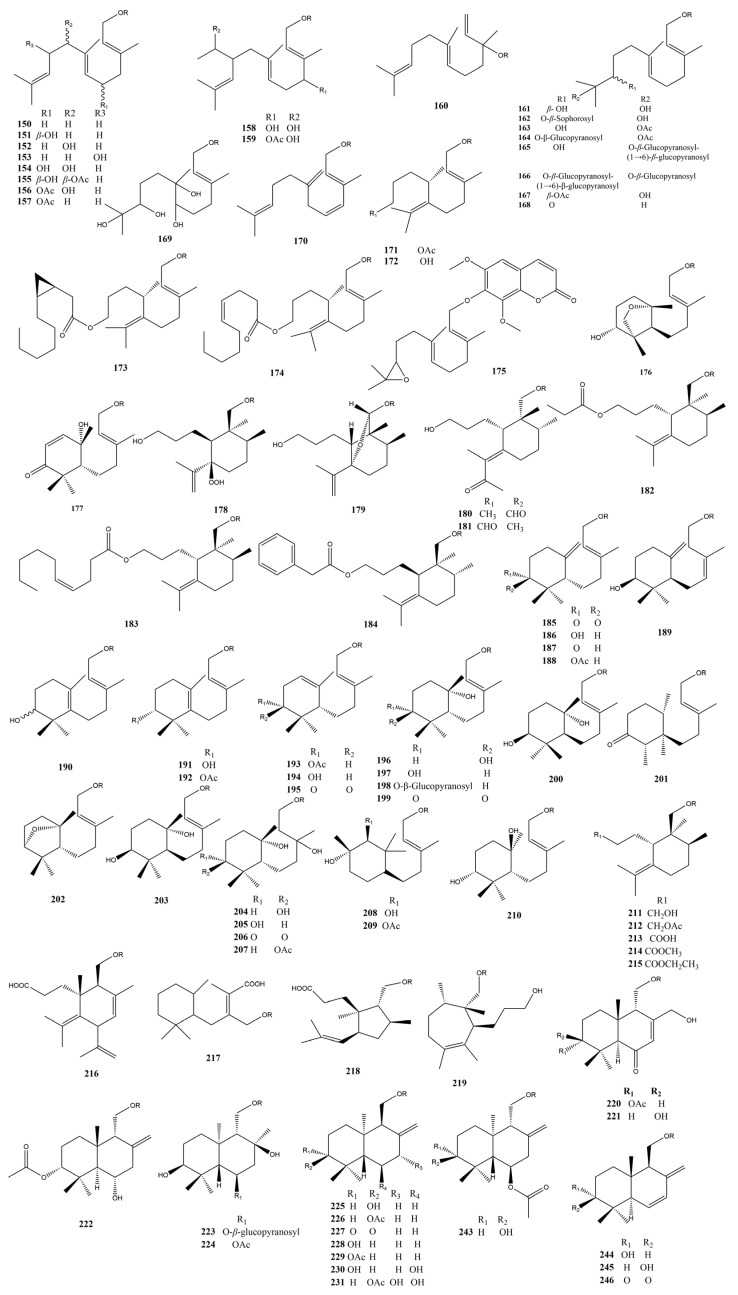

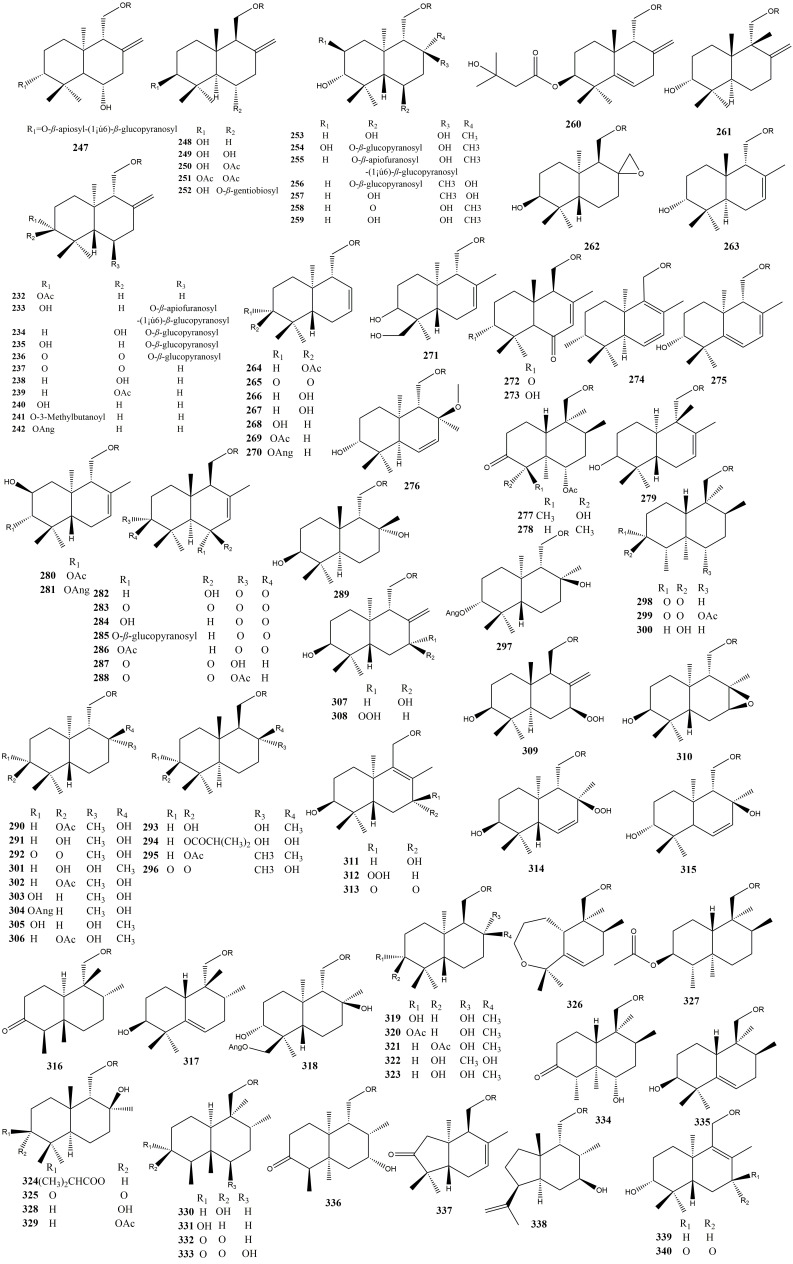

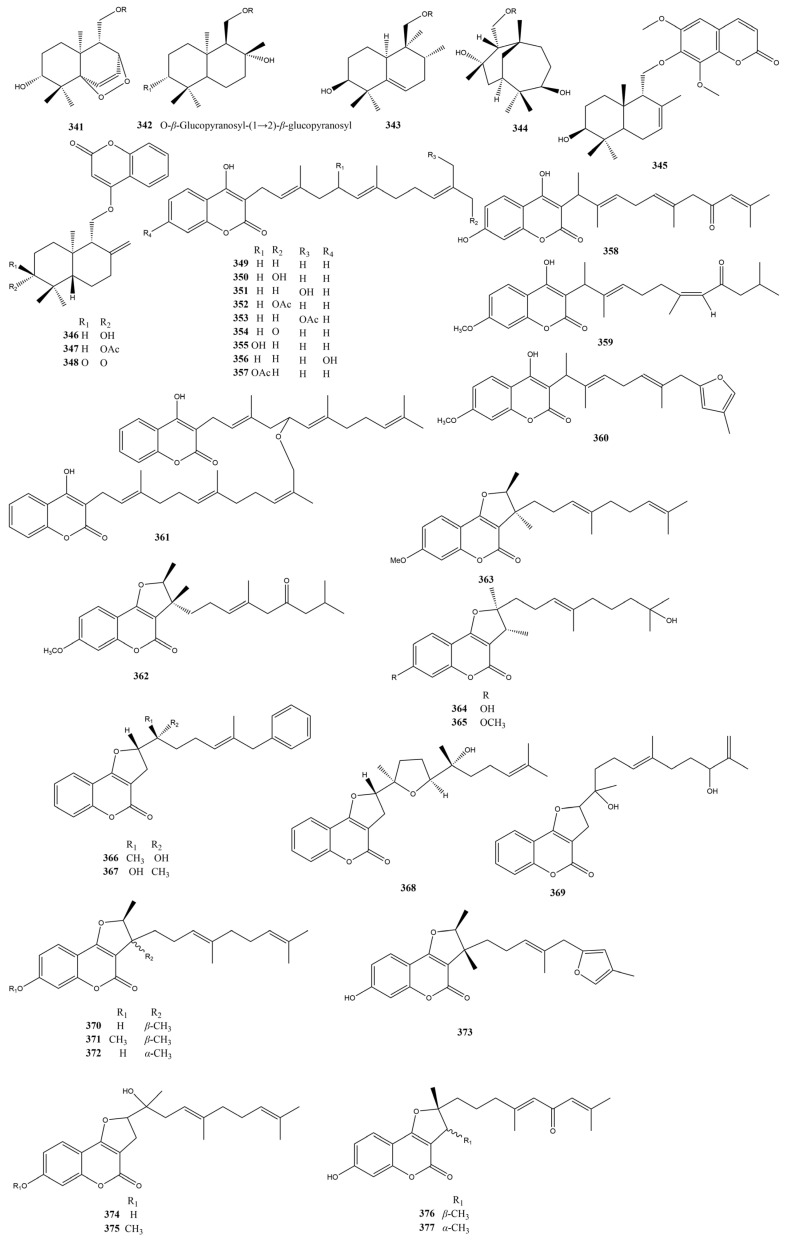

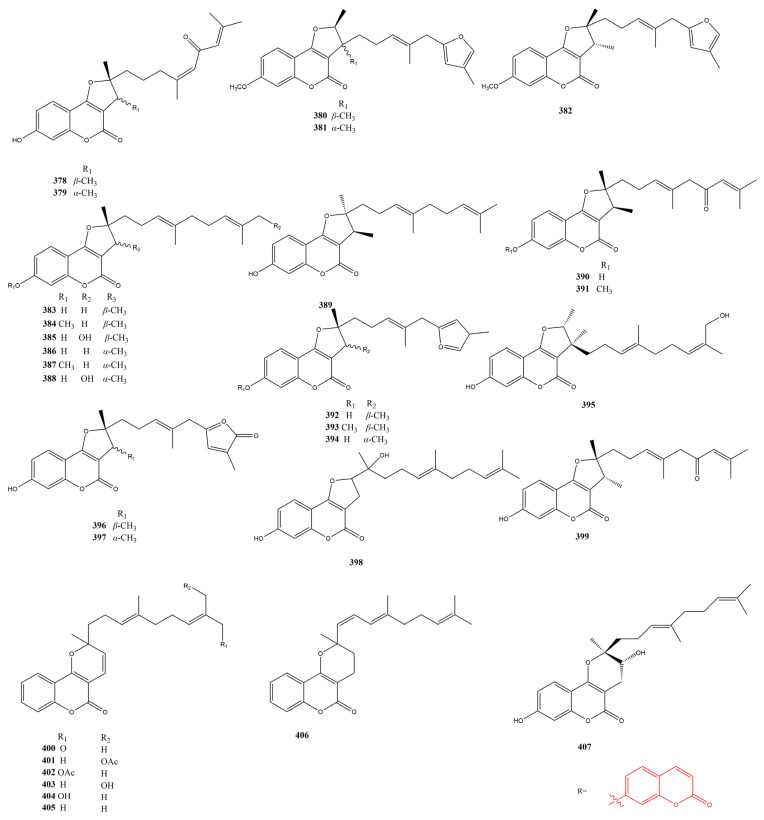
Sesquiterpene coumarins in *Ferula* plants.

The names and sources of sesquiterpene coumarins (**150**–**407**) are listed in Table 2, and their chemical structures are shown in Figure 3.

### 2.3. Sesquiterpene Chromones

Sesquiterpene chromones are present relatively rarely in the genus *Ferula*, with only 16 compounds (**408**–**423**) found in *F. communis* subsp. *communis*, *F. fukanensis*, *F. ferulaeoides*, *F. pallida*, and *F. sinkiangensis*, which can be classified into the furanochromone type (**408**–**418**) and pyranochromone type (**419**–**421**). The exceptions are (±)-ferulasin (**422** and **423**), which are uncommon sesquiterpene chromones with an oxygen-containing macrocyclic framework.

**Figure 4:**
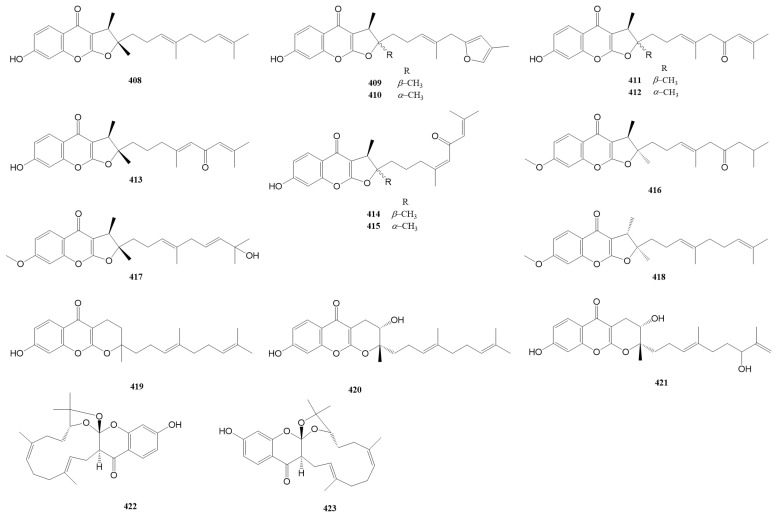
Sesquiterpene chromones from *Ferula* plants.

The names and sources of sesquiterpene chromones (**408**–**423**) are listed in Table 3, and their chemical structures are shown in Figure 4.

### 2.4. Sesquiterpene Phenylpropanoids

**Figure 5:**
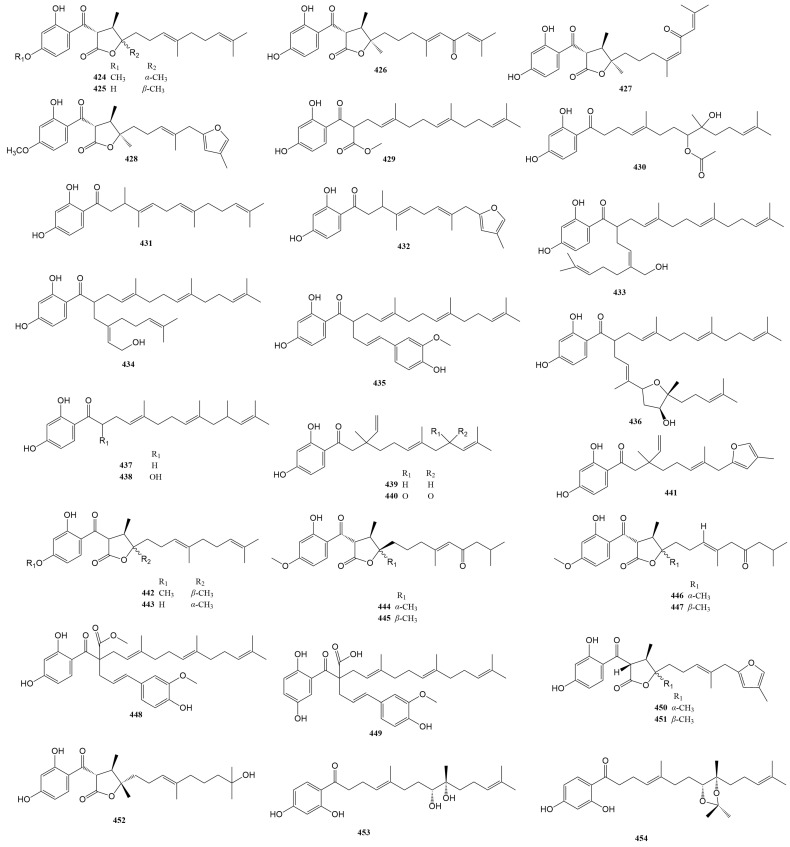
Sesquiterpene phenylpropanoids in *Ferula* plants.

Sesquiterpene phenylpropanoids (**424**–**458**) are also present only in a few *Ferula* species, such as *F. fukanensis*, *F. ferulaeoides*, *F. pallida*, *F. sinkiangensis*, and *F. seravschanica.* Their structures vary mainly in the sesquiterpene moiety, including the types and positions of substituents, stereoisomerism, etc., whereas the phenylpropanoid moiety often loses one carbon. The names and sources of compounds **424**–**458** are listed in Table 4, and their chemical structures are shown in Figure 5.

## 3. Biosynthetic Pathways

**Figure 6:**
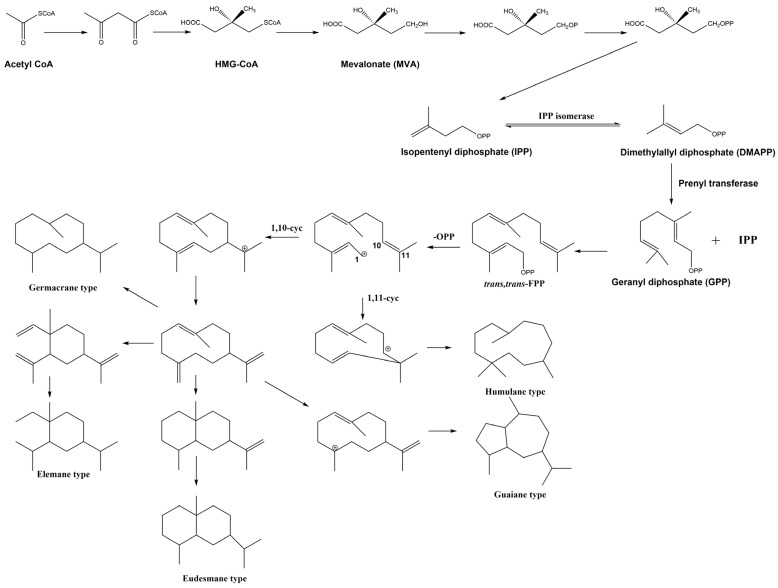
Biosynthesis pathways of the typical sesquiterpene skeletons in *Ferula* [202,203].

Sesquiterpenes are synthesized in plants through complex biosynthetic pathways, which involve several enzymatic reactions and intermediates. Sesquiterpene biosynthesis typically begins with the isopentenyl diphosphate (IPP) and dimethylallyl diphosphate (DMAPP) precursors, which are common to all terpenoids. These precursors are generated through the mevalonate (MVA) pathway or the 2-C-methyl-D-erythritol 4-phosphate (MEP) pathway. Once IPP and DMAPP are synthesized, they serve as the building blocks for sesquiterpene biosynthesis. IPP and DMAPP are condensed to form geranyl diphosphate (GPP), which contains ten carbon atoms. Then farnesyl diphosphate (FPP), which contains fifteen carbon atoms, is formed by the condensation of two molecules of IPP and GPP. FPP is a precursor to various sesquiterpenes, and it undergoes further modifications and cyclization reactions. These cyclization reactions create diverse sesquiterpene skeletons with different ring structures. After the initial cyclization step, the sesquiterpene skeleton may undergo rearrangement or further modification by various enzymes. This step introduces functional groups and structural diversity into the sesquiterpenes. After the sesquiterpenes are synthesized, they may undergo additional enzymatic modifications, such as glycosylation, acylation, or oxidation. These modifications can alter their solubility, stability, and biological activities. The biosynthesis pathways of the typical sesquiterpene skeletons in *Ferula* are shown in Figure 6.

**Figure 7:**
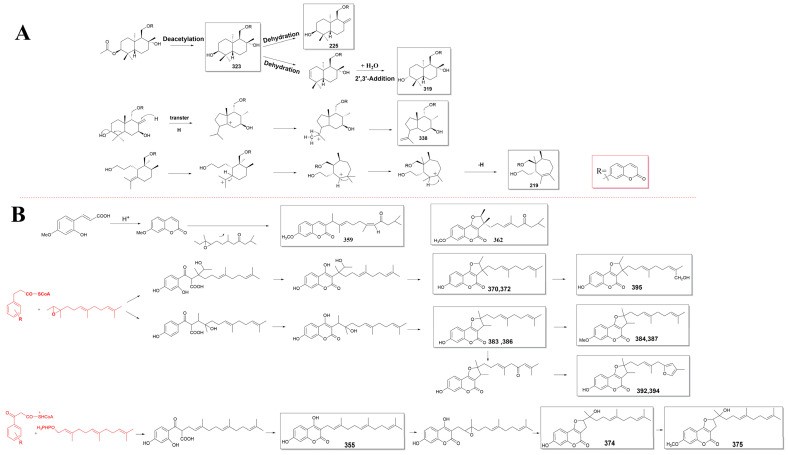
Biosynthesis pathways of different types of sesquiterpene coumarins. (**A**) The biosynthesis pathways of type I sesquiterpene coumarins [112,128,204]. (**B**) The biosynthesis pathways of type III sesquiterpene coumarins [143,179]. The biosynthesis pathways of sesquiterpene chromones [115,143,195] and sesquiterpene phenylpropanoids [179,198,199,201] are shown in Figure 8 and Figure 9, respectively.

The scaffold of sesquiterpene coumarins is formed through the dehydration condensation of the coumarin unit and the sesquiterpene moiety via an ether bond. The biosynthesis pathways of type Ib sesquiterpene coumarins, e.g., **219** [128], type Ic sesquiterpene coumarins, e.g., **225**, **319**, **323**, and **338** [112,204]; type IIIa sesquiterpene coumarins, e.g., **355** and **359** [143,179]; and type IIIb sesquiterpene coumarins, e.g., **362**, **370**, **372**, **374**, **375**, **383**, **384**, **386**, **387**, **392**, **394**, and **395** [143,179], are shown in Figure 7.

## 4. Bioactive Properties

### 4.1. Antibacterial Effects

Antibiotics, such as penicillin, tetracycline, ciprofloxacin, are a common class of antibacterial agents, which are specifically designed to target and kill or inhibit the growth of bacteria. Natural compounds can be used alongside antibiotics as complementary treatments, potentially enhancing the overall effectiveness of the treatment.

The antibacterial effects of sesquiterpenes and sesquiterpene derivatives from *Ferula* have been extensively studied. In 2001, Tamemoto et al. [64] isolated the daucane sesquiterpene ferutinin (**18**) from *F. kuhistanica*, which showed strong activity against Gram-positive bacteria, including *Staphylococcus aureus*, *S. epidermidis*, *Enterococcus faecalis*, and *Bacillus subtilis*, but did not exhibit activity against Gram-negative bacteria. Among them, the MIC values of ferutinin (**18**) were similar to those of the standard antibiotics ampicillin and chloramphenicol. In another study, Ibraheim et al. [205] reported that ferutinin (**18**), teferin (**21**), and teferidin (**17**) from *F. hermonis* showed strong activity against methicillin-resistant *S. aureus* (MRSA) (MIC, <0.39, 1.56, and 0.78 μg/mL, respectively), *B. subtilis* (MIC: <0.39, 1.56, and <0.39 μg/mL, respectively), *Mycobacterium tuberculosis* (MIC: 2, 8, and 0.69 μg/mL, respectively), and BCG (MIC: 1.56, 6.25, and 3.125 μg/mL, respectively). In addition, the enhancing effect of ferutinin (**18**) on four antitubercular drugs, i.e., rifampin, isoniazid, streptomycin, and ethionamide was tested against *M. smegmatis*. Ferutinin (**18**) showed higher activity (MIC, 10 μg/mL) than rifampin and streptomycin (MIC: each at 20 μg/mL) in the agar dilution assay, and the combination of ferutinin (**18**) with the anti-tuberculosis drugs isoniazid and ethionamide resulted in enhancing the effect of the antimycobacterial activity in the checkerboard method, while the combination with rifampicin or streptomycin did not exhibit this effect [206].

As another structural type of sesquiterpenes, the new guaianolides diversolide A (**103**), diversolide D (**106**), and diversolide F (**108**) from *F. diversivittata* showed moderate antibacterial activities against *S. aureus* (ATCC 29737) and *Escherichia coli* (ATCC 8739), with MIC values ranging from 40 to 80 μg/mL, while the MIC values of the gentamycin positive control were 18 μg/mL and 8 μg/mL, respectively [49].

In 2004, the sesquiterpene coumarin ferulenol (**349**) and its three derivatives were investigated for their antimycobacterial activity against four strains of fast-growing *Mycobacterium* species: *M. fortuitum*, *M. phlei*, *M. aurum*, and *M. smegmatis*. Ferulenol (**349**) showed the most promising activity with IC_50_ values of 0.5–2 μg/mL, which was superior to the therapeutically used antimycobacterials isoniazid and ethambutol (IC_50_: 0.5–8 μg/mL and 0.5–4 μg/mL, respectively) [207]. Ferulenol (**349**) showed stronger activity (MIC: each at 0.63 μg/mL) against the above Gram-positive bacteria, and it exhibited potent activity against *Mycobacterium* organisms (MIC: each at 1.25 μg/mL). In contrast, ferchromone (**420**) proved to be less active [208].

By the disk diffusion method, umbelliprenin (**150**) was found to have the highest activity against *B. subtilis*, *B. cereus*, *E. coli*, *Salmonella typhi*, *Klebsiella ponumoniae*, *S. aureus*, and *S. epidermilis* at a concentration of 500 μg/mL [209]. Liu et al. [116,200] discovered a series of sesquiterpene phenylpropanoids and sesquiterpene coumarins from *F. ferulioides* with strong antibacterial properties against multidrug-resistant (MDR) *S. aureus* strains, including ATCC25923, RN4220, SA1199B, XU212, EMRSA15, and EMRSA16. Among them, 8,9-oxoisopropanyldshamirone (**454**) and ferulaeolactone A (**452**) displayed better antibacterial properties than some of the controls in the above strains, i.e., norfloxacin, tetracycline, erythromycin, and oxacillin. The sesquiterpene phenylpropanoid (6*E*)-1-(2,4-dihydroxyphenyl)-3,7,11-trimethyl-3-vinyl-6,10-dodecadien-1-one (**439**) and the sesquiterpene coumarin 2,3-Dihydro-7-hydroxy-2S*,3R*-dimethyl-2-[4,8-dimethyl-3(*E*),7-nonadienyl]-furo[3,2-*c*]coumarin (**383**) from *F. heuffelii* could significantly inhibit *S. epidermidis* (MIC: 11.2 and 5.2 μM, respectively) and *Micrococcus luteus* (MIC: 22.5 and 10.5 μM, respectively) growth. Moreover, (6*E*)-1-(2,4-dihydroxyphenyl)-3,7,11-trimethyl-3-vinyl-6,10-dodecadien-1-one (**439**) could also inhibit *B. subtilis* growth, with an MIC value of 11.2 μM [210]. Sun et al. [211] discovered two sesquiterpene phenylpropanoids from *F. ferulioides* using TLC-bioautography-directed isolation. Both compounds showed significant antibacterial activities against five tested strains, especially the MDR strains XU212 and SA1199B, and the MIC values of the two compounds (16 μg/mL and 1 μg/mL, 8 μg/mL and 2 μg/mL, respectively) were lower than the standard antibiotic norfloxacin (32 μg/mL and 8 μg/mL, respectively). Ferulsinaic acid (**218**) was found to have strong antibacterial activity against Gram-positive strains (*B. cereus* and *S. aureus*) and Gram-negative strains (*Serratia* sp., *Pseudomonas* sp., and *E. coli*), which was comparable to the reference antibiotics ampicillin and amoxillin [145].

In 2007, Shahverdi et al. [212] proved that galbanic acid (**213**) could enhance the antibacterial activity of penicillin G and cephalexin against *S. aureus*. The MIC of penicillin G alone was 64 μg/mL, while the MIC of a combination of penicillin and galbanic acid (**213**) was reduced to 1 μg/mL. In the meanwhile, the MIC of cephalexin decreased from 64 μg/mL to 1 μg/mL when used in combination with galbanic acid (**213**). In 2009, Bazzaz et al. [213] proved that galbanic acid (**213**) could enhance the activity of methicillin, tetracycline, and ciprofloxacin against isolates of *S. aureus*. The MIC of methicilin, tetracycline, and ciprofloxacin decreased from 10–80 μg/mL, 40-80 μg/mL, and 10–20 μg/mL to less than 1.25 μg/mL when used in combination with galbanic acid (**213**). The class A *β*-lactamase is one of the main causes of *β*-lactam antibiotic resistance. Umbelliprenin (**150**) and galbanic acid (**213**) showed potent inhibitory activity (IC_50_: 54 ± 2.9 μM and 47 ± 3.1 μM, respectively) against class A *β*-lactamase, and the IC_50_ of the positive control, clavulanic acid, was 24.1 ± 2.1 μM. Moreover, the average MIC of penicillin G alone was 244.2 ± 12.3 μM, while the average MIC of penicillin–umbelliprenin and penicillin–galbanic acid were 21.3 ± 4.3 μM and 18.2 ± 5.6 μM, respectively, which was a significant decrease from the MIC of penicillin G. The results indicate that umbelliprenin (**150**) and galbanic acid (**213**) may be good substitutes for clavulanic acid to combat infections caused by *S. aureus* resistance [214]. Galbanic acid (**213**) appears to exert its antibacterial activity by the regulation of drug resistance.

In 2014, Dastan et al. [158] reported that 4′-hydroxy kamolonol acetate (**277**) and kamolonol (**334**) from *F. pseudalliacea* displayed antibacterial activity against *Heliobacter pylori* and *S. aureus* (MIC: each at 64 μg/mL). Later in 2016, they investigated the antibacterial effect of another six sesquiterpene coumarins from *F. pseudalliacea* against seven bacterial strains, including *S. aureus*, *B. cereus*, *E. faecium* (vancomycin-resistant clinical strain*)*, *K. pneumonia* (clinical strain), *P. aeruginosa* (clinical strain), *Helicobacter pylori*, and *E. coli*. All compounds were effective (MIC: 64-128 μg/mL) against *S. aureus*, except for farnesiferol B (**186**). Fekrynol acetate (**212**) and methyl galbanate (**214**) showed significant activity against *E. faecium*, with MIC values of 128 and 64 μg/mL, respectively, while the MIC value of the chloramphenicol control was 32 μg/mL. Ethyl galbanate (**215**) and kamonolol acetate (**278**) showed strong activity against *H. pylori*, with MIC values of 64 and 128 μg/mL, respectively [215]. The MIC values of these compounds against different bacterial strains are listed in Table 5.

### 4.2. Antifungal Effects

Over the past several decades, there has been a significant rise in the number of human fungal infections, particularly those affecting the skin and mucosal surfaces. These infections are most common in tropical and subtropical regions and are mostly caused by *Candida* sp. and dermatophytes [216]. According to research conducted by Al-Ja’fari et al. [12], ferutinin (**18**) and teferidin (**17**) from the rhizome and roots of *F. hermonis* displayed antifungal activity in vitro. The results of determining the minimal fungicidal concentration (MFC) and MIC of both substances showed that ferutinin (**18**) had greater antifungal activity than teferidin (**17**). Especially in *Tricophyton mentagrophytes*, their MIC and MFC values ranged from 8 to 256 mg/mL.

### 4.3. Antiparasitic Effects

Iranshahi et al. [122] and Bashir et al. [151] evaluated the inhibitory activity of sesquiterpene coumarins extracted from *F. szowitsiana* and *F. narthex* Boiss against *Leishmania major*. The results showed that umbelliprenin (**150**) and conferol (**267**) displayed potent antileishmanial activity (IC_50_: 4.9 and 11.5 μg/mL, respectively). Dastan et al. [121] determined the in vitro antiplasmodial activity of compounds extracted from *F. pseudalliacea* against the *Plasmodium falciparum* strain, K1. The results indicated that kamolonol acetate (**299**) and methyl galbanate (**214**) showed moderate antiplasmodial activity, with IC_50_ values of 16.1 and 7.1 μM, respectively, whereas the IC_50_ value of the positive control, artemisin, was 0.004 μM.

### 4.4. Antiviral Effects

H1N1, also known as swine flu, is a subtype of the influenza A virus. In 2009, it attracted worldwide attention when a new H1N1 strain emerged and caused a global pandemic. It was found that some sesquiterpene coumarins of *Ferula* species were active against H1N1. Lee et al. [10] discovered that 5′*S*-hydroxyumbelliprenin (**151**), 8′-acetoxy-5′*S*-hydroxyumbelliprenin (**155**), methyl galbanate (**214**), galbanic acid (**213**), farnesiferol C (**202**), farnesiferol A (**228**), conferol (**267**), ligupersin A (**273**), and *epi*-conferdione (**272**) isolated from *F. assa-foetida* displayed significant antiviral activity against H1N1 (IC_50_: 0.26–0.86 μg/mL), which was more effective than amantadine (IC_50_: 0.92 μg/mL). Li et al. [30] found that Sinkiangenorin E also had a significant inhibitory effect on H1N1. These findings indicate that sesquiterpene coumarins might be potential lead compounds for new drugs to treat H1N1 viral infection. In addition, the sesquiterpene coumarin kellerin (**321**) showed an antiviral effect against herpes virus type 1 (HSV-1) by the plaque-reduction assay. It could dramatically reduce the viral titre of the HSV-1 DNA viral strain KOS at concentrations of 10, 5, and 2.5 µg/mL and considerably lessen its cytopathic effects [217].

### 4.5. Antioxidative Effects

Oxidative stress refers to the imbalance between the antioxidative defense system and the production of oxidants (free radicals). The accumulation of oxidized lipids plays an important role in the incidence of many diseases such as diabetes, cancer, aging, etc. Therefore, compounds that reduce or prevent the production of oxidative products can be used to treat these diseases [218].

In a study conducted by Raafat and El-Lakani [219], it was observed that the administration of ferutinin (**18**), a daucane-type sesquiterpene ester, significantly reversed the decreasing trend of the expression of the antioxidant enzyme catalase observed in diabetic mice. In addition, for the first time, it described the antioxidant property of ferutinin (**18**) on diabetes-related neuropathic pain, indicating that 1.6 mg/kg of ferutinin (**18**) could reduce thermal hyperalgesia and tactile allodynia. At 500 and 1000 μg/kg mice body weight, ferutinin (**18**) could significantly upregulate the gene expression of superoxide dismutase (SOD), catalase (CAT), and glutathione peroxidase (GPx) in liver and kidney tissues, which are known to resist cellular oxidative stress. At the same concentration, it could also significantly decrease the lipid peroxidation in mice liver tissues [220]. An analog of ferutinin (**18**), 2*α*-acetyl ferutinin (**19**) could rapidly reduce the mRNA levels of several intracellular antioxidative enzymes, such as catalase, Mn-superoxide dismutase (SOD2), nuclear factor erythroid 2-related factor 2 (NRF2), peroxiredoxin (PRDX1), and thioredoxin (TRX) between 6 and 12 h, and it could also significantly induce intracellular glutathione (GSH) depletion in a time- and concentration-dependent manner [32]. In addition, the daucane esters teferidin (**17**), ferutinin (**18**), and teferin (**21**) from *F. hermonis* showed strong 2,2-diphenyl-1-(2,4,6-trinitrophenyl)hydrazyl (DPPH) radical scavenging activity, with IC_50_ values of 17.3, 13.2, and 11.5 μM, respectively, comparable to the positive control of ascorbic acid (12.5 μM). The significant increase in free radical scavenging activity is associated with an increase in the number of hydroxyl groups [205].

Umbelliprenin (**150**) is the first synthesized sesquiterpene coumarin in *Ferula*. In a study on the antigenotoxicity effects of umbelliprenin (**150**) on human peripheral lymphocytes exposed to oxidative stress [221], although umbelliprenin (**150**) showed no scavenging activity (4%), the protective activity of umbelliprenin (**150**) (10-400 mM) against DNA damage induced by 25 mM H_2_O_2_ increased in a concentration-dependent manner. There was no significant difference between umbelliprenin (**150**) and ascorbic acid (positive standard) when the concentration exceeded 50 mM. Kamolonol acetate (**299**) is also a sesquiterpene coumarin extracted from *F. pseudalliacea* with potent antioxidant activity. It displays strong DPPH radical scavenging activity, with an EC_50_ value of 65.29 ± 5.6 μM, which is similar to that of the positive control, butylatedhydroxyanisole (BHA), at 59.85 ± 3.7 μM [222].

Kogure et al. [223] evaluated the antioxidative activities of several compounds isolated from *F. penninervis* and *F. pallida*, with the sesquiterpene coumarin KT23 (Pallidone A) (**359**) having moderate antioxidative properties. Compared with *α*-tocopherol (43.2%, 200 μM) as a control, KT23 (**359**) (100 μM) inhibited 16.4% of egg-yolk phosphatidylcholine liposome (EyPC liposome) peroxidation. Ferulsinaic acid (**218**) is a sesquiterpene coumarin from *F. sinaica* with a rare carbon skeleton. It was found to significantly reduce malondialdehyde (MDA) levels in *Caenorhabditis elegans*, thus attenuating lipid peroxidation. In addition, it could significantly decrease the formation of N-*ε*-carboxymethyllysine (CML), one of the advanced glycation end-products (AGEs) that is correlated with oxidative stress. These indicate the antioxidative power of ferulsinaic acid (**218**) [224]. Galbanic acid (**213**) is also a natural sesquiterpene coumarin abundantly distributed in *Ferula* species; it exhibited antioxidative activity by inhibiting DPPH and ABTS free radicals, with IC_50_ values of 180 and 60 μg/mL, respectively. In addition, galbanic acid (**213**) (62.5 μg/mL) and vitamin C (5 μg/mL), as a positive control, could significantly upregulate the expression of SOD, CAT, and GPx. The upregulation of these antioxidative genes enhances the redox state of cells; however, the potential of galbanic acid (**213**) to upregulate antioxidative enzymes is lower than that of vitamin C [225].

### 4.6. Anti-Inflammatory Effects

Inflammation is a complex biological response triggered by the immune system in response to harmful stimuli such as infections, injuries, or diseases. Many sesquiterpenes and their derivatives from *Ferula* have anti-inflammatory properties, making them valuable for promoting overall health and potentially reducing the risk of chronic diseases associated with inflammation.

Ferutinin (**18**) and teferin (**21**) exhibit anti-inflammatory effects at a dose of 100 mg/kg using the in vivo carrageenan-induced edema model, which may be caused by the antagonistic effects of histamine and/or serotonin actions, and their anti-inflammatory effects may be directly related to the degree of oxidation of the benzene ring [226].

The sesquiterpene coumarins methyl galbanate (**214**) and umbelliprenin (**150**) were reported to exert their anti-inflammatory effects by significantly inhibiting the LPS-induced production of nitric oxide (NO) and prostaglandin E_2_ (PGE_2_), leading to a decrease in the expression of inducible nitric oxide synthase (iNOS) and cyclooxygenase-2 (COX-2) [227]. In another study, the anti-inflammatory effect of umbelliprenin (**150**) was evaluated in vitro and in vivo. It displayed a significant inhibitory effect on soybean lipoxygenase (a key enzyme in the process of inflammation), with an IC_50_ value of 0.0725 μM, whereas the IC_50_ value of the positive control, caffeic acid, was 600 μM. Furthermore, it showed a significant anti-inflammatory effect (39%) in vivo, which was comparable to the positive control, indomethacin (47%), using the carrageenin mouse-paw edema model [228]. In RAW264.7 cells stimulated by lipopolysaccharide (LPS)/interferon-γ (IFN-γ), Kohno et al. [229] found that methyl galbanate (**214**) significantly reduced NO production. In the presence of methyl galbanate (**214**), the mRNA expression of iNOS stimulated by LPS/IFN-γ was reduced to 52% of the levels found with LPS/IFN-γ induction alone.

Kellerin (**321**) is the major constituent (1.5%, *w*/*w*) of *F. sinkiangensis*, and its anti-inflammatory mechanism is to inhibit the mRNA expression of inflammatory cytokines such as NO, tumor necrosis factor-*α* (TNF-*α*), COX-2, interleukin-6 (IL-6), and interleukin-1*β* (IL-1*β*) [190]. Zhang et al. [190] found that kellerin (**321**) could transform microglia from a pro-inflammatory M1 phenotype into an anti-inflammatory M2 phenotype, and thus alleviate cognitive impairment in mice. In another research, kellerin (**321**) was found to decrease the levels of pro-inflammatory cytokines, inhibit the NF-κB signaling pathway, and reduce ROS production and NADPH oxidase activity to exert neuroprotective effects [27].

Motai et al. reported six new sesquiterpene coumarins from *F fukanensis*, four of which (fukanefuromarin H–K) (**385**, **388**, **396**, and **397**) showed NO-inhibitory activities, with IC_50_ values of 11.1-55.6 μM. In addition, fukanefuromarin H (**385**) and fukanefuromarin K (**397**) could inhibit the gene expression of iNOS, IL-6, and TNF-*α* [156]. The sesquiterpene coumarins ferubungeanol B (**308**) (IC_50_ 23.6 μM) and samarcandin acetate (**290**) (IC_50_ 25.6 μM) from *F. bungeana* were found to have a strong inhibitory effect on NO production in LPS-induced BV-2 microglia compared with the positive control, minocycline (IC_50_ 25.6 μM) [137]. Episamarcandin acetate (**295**) from *Ferula sinkiangensis* was also found to exert its anti-inflammatory activity by significantly decreasing NO production, with an IC_50_ value of 2.3 μM, and inhibiting TNF-*α*, IL-1β, and IL-6 expression [149].

### 4.7. Antitumor Effects

In the past few decades, the cytotoxicity of sesquiterpenes and sesquiterpene derivatives from the *Ferula* species, especially sesquiterpene coumarins, has been studied extensively. These compounds have shown significant cytotoxicity against various tumor cell lines, including HCT116 and HT-29 human colon cancer cells; AGS, BGC-823, and MGC-803 human gastric cancer cells; M4Beu human melanoma cells; BxPC3, PANC-1, and Capan-1 human pancreatic cancer cells; HeLa human cervical cancer cells; and MCF-7 and MDA-MB-231 human breast cancer cells. The IC_50_ values of these compounds against different cancer cell lines in vitro are listed in Table 6.

Umbelliprenin (**150**) is one of the most widely studied sesquiterpene coumarins with antitumor potential. Promoting tumor cell apoptosis is one of the important mechanisms in antitumor therapy. Researchers discovered that umbelliprenin (**150**) could promote apoptosis in tumor cells by annexin V-FITC/PI staining. In the meanwhile, umbelliprenin (**150**) activated caspase-3, -8, and -9 and the proapoptotic protein Bax and reduced the expression of the antiapoptotic protein Bcl-2, caspase-3, -8, and -9, and the proapoptotic protein Bax and reduced the expression of the antiapoptotic protein Bcl2 [230,231], which promoted apoptosis in the Jurkat T-CLL and Raji B-CLL cell lines in a time- and dose-dependent manner [232]. In addition, it could activate the mitochondrial apoptotic pathway and lead to apoptosis of the cancer cells by decreasing the mitochondrial membrane potential, enhancing the P53, P27, P16, and Rb protein expression and diminishing the expression of the proteins of cyclin E, cyclin D, Cdk4, and Cdk6 as well as cell cycle arrest in the G0/G1 phase [233]. Apart from this, umbelliprenin (**150**) could attenuate cell migration through the Wnt signaling pathway by decreasing the expression levels of Wnt-2, β-catenin, GSK-3β, p-GSK-3β, survivin, and c-myc [193]. In another study, umbelliprenin was found to induce cytoprotective autophagy by reducing the phosphorylation levels of AKT and mTOR and blocking the Akt signaling pathway [230]. In brief, umbelliprenin (**150**) could exert its antitumor property by inducing apoptosis and autophagy, inhibiting the cell cycle, and attenuating the migration and invasion of cancer cells. In an in vivo study, a double-stage carcinogenicity assay of mouse skin tumors was performed to investigate the cancer chemopreventive activity of umbelliprenin (**150**). The results showed that mice treated with umbelliprenin (**150**) together with peroxynitrite (initiator)/TPA (promoter) had delayed papillary tumor formation, with effects comparable to those of the curcumin control. Furthermore, the tumor development pattern was slower in umbelliprenin-treated mice compared with curcumin treatment. Thus, umbelliprenin (**150**) may be a potential cancer chemopreventive agent [234].

Galbanic acid (**213**) is another extensively studied sesquiterpene coumarin. Kim et al. [235] revealed the potential molecular mechanism of galbanic acid (**213**) in overcoming chemotherapy resistance in drug-resistant lung cancer. As an effective TNF-related apoptosis-inducing ligand (TRAIL) sensitizer, galbanic acid (**213**) enhanced TRAIL-induced cell apoptosis by inhibiting multidrug resistance 1 (MDR1) and activating caspase and death receptor 5 (DR5) in cisplatin-resistant H460/R non-small-cell lung cancer cells. Galbanic acid (**213**) induced tumor cell-cycle arrest at G_1_, which is associated with the inhibition of the cyclin/cyclin-dependent kinase (CDK)4/6 pathway, particularly cyclin D_1_. [236]. It also inhibited tumor cell metastasis. Neoangiogenesis and the activation of matrix metalloproteinases (MMPs) play a crucial role in tumor generation and metastasis. Neovessels are formed during tumor generation and metastasis [237], so the inhibition of angiogenesis may promote cancer cell death [238]. Kim et al. [239] reported that galbanic acid (**213**) reduced the number of blood vessels in tumor cells by more than 40%, significantly reduced the proliferation of vascular endothelial growth factor-(VEGF)-induced human umbilical-vein endothelial cells (HUVECs), and inhibited VEGF-induced migration and tube formation in HUVECs. It was shown to have an inhibitory effect on tumor-induced angiogenesis. MMPs are capable of degrading the vast majority of proteins in the extracellular matrix and disrupting the extracellular matrix and basement membrane barriers of tissues, which play a crucial role in the invasive and metastatic process of cancer cells [240]. Thus, inhibiting the activity of MMPs is an effective strategy to block the migration of tumor cells. Studies have demonstrated that galbanic acid (**213**) can inhibit the activity and expression of MMP2 and MMP9 [241]. Hypoxia-inducible factor (HIF) is a transcription factor that regulates the expression of genes involved in the regulation of hypoxic mechanisms (e.g., angiogenesis or apoptosis) as well as tumor growth, invasion, and metastasis [242]. Hypoxia in tumors can stimulate and induce HIF-1α and HIF-2α protein expression [243]. EGFR-MAPK is an important signaling pathway with regulatory effects on HIF-1α expression [244]. Syeda et al. [245] found that galbanic acid (**213**) downregulated HIF-1α and HIF-1β mRNA expression under both hypoxic and normoxic conditions, and it had an inhibitory effect on HIF-1 activation. Under normoxic conditions, it shortened the half-life of the EGFR (HIF-1 downstream genes) and promoted EGFR degradation to inhibit HIF activation. Meantime, it inhibited HIF-1α accumulation in A549 and OVCAR-3 cells by suppressing the EGFR/HIF-1α signaling pathway [244].

**Table 6:** The IC_50_ values of sesquiterpenes and sesquiterpene derivatives against different cancer cell lines.

Names	No.	Cell Lines	IC_50_ (μM)	References
8-*O*-Acetyl-sinkiangenorin F	**209**	AGS	62.7 ± 2.5	[81]
Coladin	**232**	HCT116	3.7 ± 1.5	[75]
		HT-29	5.4 ± 1.2	[75]
Conferol	**267**	COLO205	11.19 ± 0.68	[246]
Conferone	**265**	COLO205	27.63 ± 0.69	[246]
		MCF-7	34.02 ± 0.68	[246]
Episamarcandin	**305**	AGS	83.8 ± 1.4	[128]
Farnesiferol A	**228**	HeLa	20 ± 0.2	[189]
Farnesiferol C	**202**	HeLa	25 ± 0.8	[189]
		AGS	101.6 ± 1.3	[128]
Fekolone	**187**	AGS	75.4 ± 2.1	[128]
Fekrynol	**211**	HeLa	35 ± 0.6	[189]
		MGC-803	49 ± 0.8	[189]
		AGS	20 ± 0.5	[189]
Fekrynol acetate	**212**	HeLa	25 ± 0.6	[189]
		MGC-803	28 ± 1.2	[189]
(+)-Ferulasin	**422**	PANC-1	2.24 ± 0.83	[195]
		CFPAC-1	6.12 ± 0.52	[195]
		SW1990	11.77 ± 1.57	[195]
		Capan-2	8.57 ± 0.59	[195]
(-)-Ferulasin	**423**	PANC-1	0.92 ± 0.12	[195]
		CFPAC-1	19.13 ± 2.99	[195]
Feselol	**268**	COLO205	38.41 ± 0.8	[246]
		MCF-7	35.95 ± 1.29	[246]
Galbanic acid	**213**	HeLa	43 ± 2.0	[189]
		MCF-7	56.65 ± 1.4	[225]
		MDA-MB-231	48.75 ± 1.16	[225]
4′-Hydroxy kamolonol acetate	**277**	HeLa	4.5 ± 0.1	[158]
13-Hydroxyfeselol	**271**	HCT116	34.1 ± 2.3	[75]
		HT-29	35.4 ± 4.0	[75]
Kamolonol	**334**	HeLa	3.8 ± 0.1	[158]
Kellerin	**321**	HeLa	37 ± 1.8	[189]
		MCF-7	18.24 ± 0.12	[246]
Lehmannolol	**300**	HeLa	42 ± 0.9	[189]
		AGS	26.0 ± 0.9	[128]
Lehmannolone	**298**	HeLa	81.1 ± 1.4	[128]
Mogoltadone	**227**	COLO205	31.71 ± 0.15	[246]
		MCF-7	30.45 ± 0.6	[246]
		K-562	21.11 ± 0.85	[246]
		HepG2	23.06	[247]
Polyanthinin	**226**	HeLa	28 ± 0.4	[189]
		MGC-803	45 ± 0.9	[189]
		AGS	45 ± 0.9	[189]
Sinkiangenol E	**222**	HeLa	16 ± 0.8	[189]
Sinkiangenone A	**448**	MGC-803	45.05 ± 3.09	[21]
		AGS	48.13 ± 0.87	[21]
Sinkiangenone B	**449**	MGC-803	18.89 ± 1.32	[21]
		AGS	16.15 ± 0.14	[21]
Sinkiangenorin D	**219**	HeLa	20.4 ± 1.3	[128]
		AGS	104.8 ± 1.2	[128]
		K562	81.1 ± 1.0	[128]
Sinkiangenorin E	**344**	AGS	12.7 ± 2.5	[30]
Sinkiangenorin F	**208**	AGS	27.1 ± 1.4	[81]
Sinkianone	**201**	HeLa	77.9 ± 0.7	[128]
Umbelliprenin	**150**	AGS	11.74 ± 1.33	[193]
		BGC-823	24.62 ± 2.45	[193]
		M4Beu	12.4	[232]
		BxPC3	45.15 ± 2.57	[230]
		PANC-1	47.13 ± 5.13	[230]

**Figure 10:**
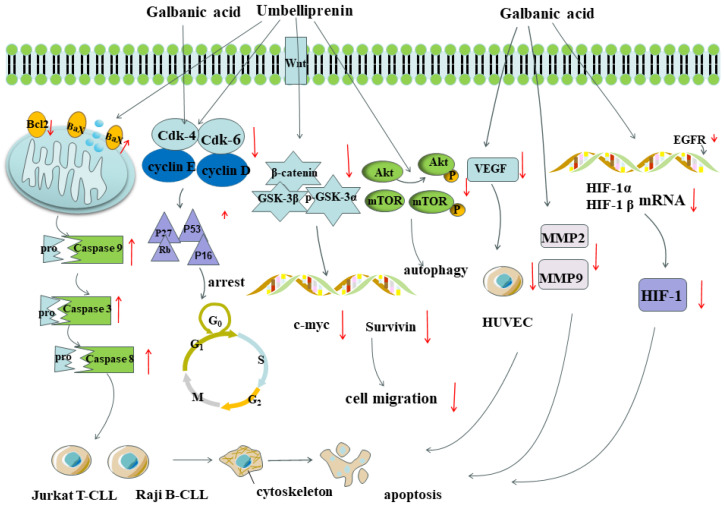
The anticancer mechanisms of umbelliprenin (**150**) and galbanic acid (**213**). MMPs (matrix metalloproteinases), HUVEC (human umbilical vein endothelial cell), HIF (hypoxia-inducible factor), VEGF (vascular endothelial growth factor), EGFR (epithelial growth factor receptor), AKT (protein kinase B). “Red arrow” respresent upregulation or downregulation.

The anticancer mechanisms of umbelliprenin (**150**) and galbanic acid (**213**) are shown in Figure 10.

### 4.8. Anti-Acetylcholinesterase Effects

Dastan et al. [163] evaluated the acetylcholinesterase (AChE) inhibitory activity of kamonolol acetate (**278**) from *F. pseudalliacea*. The results revealed that AChE was suppressed by kamonolol acetate (**278**), with an IC_50_ value of 63.9 μM. Moreover, they proved that kamonolol acetate (**278**) inhibited AChE in the mixed-type model through kinetics together with molecular modeling studies. The findings suggested that kamonolol acetate (**278**) might be a potential lead compound for designing AChE inhibitors.

### 4.9. Antidiabetic Effects

Amin et al. [248] conducted antiglycosylation tests on components isolated from the *F. narthex* exudate. The antiglycation activity of the isolated constituents is composed of both oxidative and non-oxidative inhibition modes. In the bovine serum albumin (BSA)-glucose test, ligupersin A (**273**) displayed a higher activity (IC_50_: 0.41 mM) than the control, aminiguanidine (IC_50_: 1.75 mM). In the BSA-methyl glyoxal (MGO) experiment, 5′-acetoxy-8′-hydroxyumbelliprenin (**155**) showed better activity (IC_50_: 1.03 mM) than the control, aminoguanidine (IC_50_: 0.15 mM). In another study, 10′*R*-acetoxy-11′-hydroxyumbelliprenin (**167**) displayed α-glucosidase inhibitory activity, with an IC_50_ value of 0.05 mM. The results indicated that the antidiabetic activity of the *F. narthex* exudate may be related to the presence of these constituents [249].

## 5. Conclusions

Several *Ferula* species have a long history of use in traditional medicine due to their potential therapeutic properties in treating various health conditions, such as gastrointestinal disorders, respiratory issues, and inflammatory diseases. In recent years, due to its important edible and medicinal values, extensive research has been conducted on every aspect of *Ferula*, such as its geographical distribution, physiological ecology, genomics, metabolomics, taxonomy, phytoconstituents, biosynthesis, pharmacological activity, traditional uses, clinical efficacy, and industrial applications [250,251,252,253,254].

*Ferula* is known for its production of sesquiterpenes. Sesquiterpenes are a subclass of terpenes, which are natural hydrocarbons synthesized by plants, including the *Ferula* species, through the mevalonic acid pathway. Sesquiterpenes are composed of three isoprene units, giving them a 15-carbon structure. Sesquiterpenes and their derivatives have antibacterial, antifungal, and antiviral activities, which are characteristically related to plant defense mechanisms [255]. In this work, information on 454 sesquiterpenes and their derivatives from various parts of this plant, including resins, stems, aerial parts, seeds, and roots have been summarized. The specific sesquiterpenes found in *Ferula* species can vary between different plant varieties, and even within the same species, and they are influenced by factors such as environmental conditions and geographic location. These compounds not only give *Ferula* plants their unique aromas but also contribute to their potential therapeutic properties, making them of interest to researchers and practitioners in the fields of herbal medicine. Sesquiterpenes are known for their diverse biological activities, including antioxidative, antibacterial, and anti-inflammatory properties. Ferutinin (**18**), umbelliprenin (**150**), and galbanic acid (**213**) are sesquiterpenes from *Ferula* which have undergone extensive pharmacological activity research, and investigating these activities can help uncover potential treatments for a wide range of health conditions. They have also shown promise in drug discovery and development.

It should be noted that the specific biological activity of sesquiterpenes is related to their chemical structure. Researchers should understand their structure–activity relationships to design compounds with better activity, fully tap into their therapeutic potential, and develop standardized applications in medicine industries.

In summary, *Ferula* plants offer a wealth of research opportunities in fields such as phytochemistry, pharmacology, agriculture, ecology, and biotechnology. The diverse sesquiterpenes produced by *Ferula* species have the potential to yield novel drugs, making them a valuable subject of study for researchers across the globe.

## Figures and Tables

**Figure 8:**
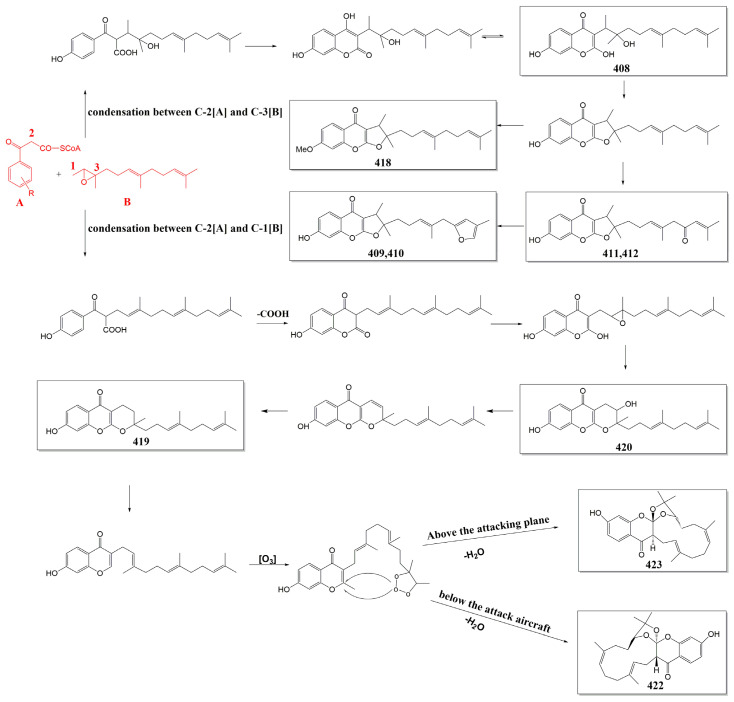
Proposed biosynthetic pathways for sesquiterpene chromones [115,143,195].

**Figure 9:**
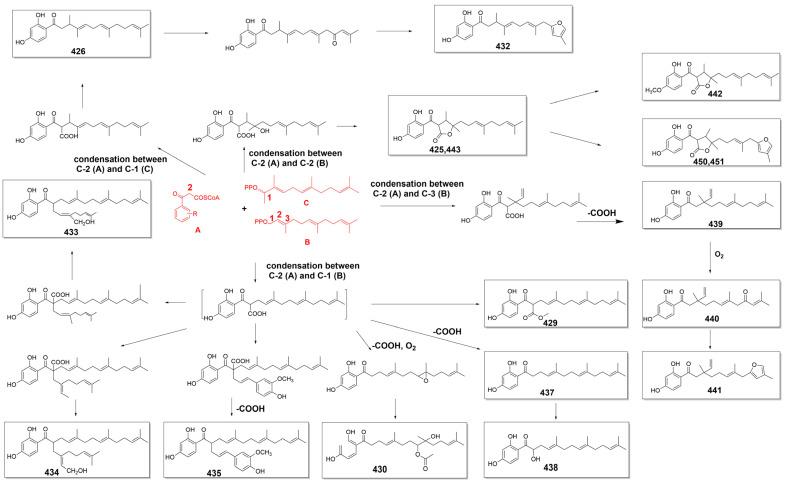
Proposed biosynthetic pathways for sesquiterpene phenylpropanoids [179,198,199,201].

**Table 1:** Sesquiterpenes in *Ferula* plants.

Names	No.	Types	Species	References
2*α*-Acetoxy-6*α*-*p*-methoxybenzoyl-10*α*-acetoxy-jaeschkeanadiol	**6**	I	*F. communis* subsp. *communis*	[31]
2*α*-Acetoxy-6*α*-*p*-methoxybenzoyl-10*α*-hydroxy-jaeschkeanadiol	**3**	I	*F. communis* subsp. *communis*	[31]
2*α*-Acetoxy-6*α*-*p*-methoxybenzoyl-10*β*-acetoxy-jaeschkeanadiol	**5**	I	*F. communis* subsp. *communis*	[31]
2*α*-Acetoxy-6*α*-*p*-methoxybenzoyl-10*β*-hydroxy-jaeschkeanadiol	**4**	I	*F. communis* subsp. *communis*	[31]
2*α*-Acetyl ferutinin	**19**	I	*F. campestris* (Besser) Grecescu	[32]
9-*O*-Acetyl-8-*O*-tigloyltovarol	**140**	V	*F. persica* Willd	[33]
Aeschkeanadiol-2-methylbutyrate	**13**	I	*F. linkii* Webb & Berthel.	[34]
8*α*-Angeloyloxy-10*β*-hydroxyslov-3-en-6,12-olide	**113**	II	*F. varia* (Schrenk) Trautv.	[35]
10-Angeloyloxy-6-p-hydroxybenzoyl-jaeschkeandiol	**20**	I	*F. communis* var. *brevifolia*	[36]
			*F. sinaica* L.	[37]
2*α*-Angeloyloxy-6-veratrate-jaechkeanadiol	**28**	I	*F. communis* subsp. *communis*	[38]
			*F. licentiana* var. *tunshanica* (Su) Shan et Q.X.Liu	[39]
14-(4′-Anisoyloxy)dauc-4,8-diene	**38**	I	*F. communis* subsp. *communis*	[40]
Badrakemonin	**143**	VI	*F. badrakema* Kos.-Pol.	[41]
Benzoylfervanol	**124**	III	*F. lycia* Boiss.	[22]
Carotdiol acetate	**41**	I	*F. linkii* Webb & Berthel.	[42]
Carotdiol veratrate	**42**	I	*F. linkii* Webb & Berthel.	[42]
Daucol	**73**	I	*F. linkii* Webb & Berthel.	[42]
10-Deangeloylpallinin	**31**	I	*F. arrigonii* Bocchieri	[43]
Decipenin D	**132**	IV	*F. penninervis* Regel et Schmalh.	[44]
7,11-Dehydrogrilactone	**114**	II	*F. arrigonii* Bocchieri	[43]
Dehydrooopodin	**138**	IV	*F. varia* (Schrenk) Trautv.	[35]
2,10-Diacetyl-8-hydroxyferutriol-6-anisate	**49**	I	*F. vesceritensis* Coss. & Durieu ex Trab.	[45]
3*α*,4*β*-Dihydroxy-5*β*H,11*α*H-eudesman-6,12-olide	**130**	IV	*F. sinaica* L.	[46]
4*β*,8*β*-Dihydroxy-6*α*-(4-hydroxy-3-methoxybenzoyl)-dauc-9-ene	**52**	I	*F. hermonis* Boiss	[47]
			*F. kuhistanica* Korovin	[48]
4*β*,8*α*-Dihydroxy-6*α*-vanilloy-loxydauc-9-ene	**54**	I	*F. kuhistanica* Korovin	[48]
Diversolide A	**103**	II	*F. diversivittata* Regel & Schmalh.-Rech.	[49]
Diversolide B	**104**	II	*F. diversivittata* Regel & Schmalh.-Rech.	[49]
Diversolide C	**105**	II	*F. diversivittata* Regel & Schmalh.-Rech.	[49]
Diversolide D	**106**	II	*F. diversivittata* Regel & Schmalh.-Rech.	[49]
Diversolide E	**107**	II	*F. diversivittata* Regel & Schmalh.-Rech.	[49]
Diversolide F	**108**	II	*F. diversivittata* Regel & Schmalh.-Rech.	[49]
Diversolide G	**109**	II	*F. diversivittata* Regel & Schmalh.-Rech.	[49]
Elaeochytrin A	**45**	I	*F. elaeochytris* Korovin	[50]
Elaeochytrin B	**50**	I	*F. elaeochytris* Korovin	[50]
Epoxy ferutinol benzoate	**80**	I	*F. hermonis* Boiss	[51]
8,9-Epoxy-ferutinin	**81**	I	*F. kuhistanica* Korovin	[48]
Epoxyjaeschkeanadiol	**78**	I	*F. hermonis* Boiss	[52]
2,3-Epoxy-jaeschkeanadiol-*p*-methoxybenzoate	**82**	I	*F. kuhistanica* Korovin	[48]
			*F. communis* L.	[53]
			*F. lancerottensis* Parl.	[54]
			*F. linkii* Webb & Berthel.	[55]
Epoxyvesceritenol	**84**	I	*F. vesceritensis* Coss. & Durieu ex Trab.	[45]
(1*R*,3*S*,8*S*)-3-Ethoxy-8-angeloyloxydauca-4-en-9-one	**70**	I	*F. hermonis* Boiss	[56]
Fercolide	**47**	I	*F. communis* subsp. *communis*	[40]
Fercomin	**39**	I	*F. communis* subsp. *communis*	[40]
			*F. licentiana* var. *tunshanica* (Su) Shan et Q.X.Liu	[57]
Ferugin	**71**	I	*F. sinaica* L.	[58]
Feruginin	**48**	I	*F. jaeschkeana* Vatke	[59,60]
Feruhermonin A	**57**	I	*F. hermonis* Boiss	[47]
Feruhermonin B	**51**	I	*F. hermonis* Boiss	[47]
Feruhermonins C	**62**	I	*F. hermonis* Boiss	[47]
Ferulactone A	**147**	others	*F. ferulaeoides* (Steud.) Korov	[61]
Ferulactone B	**148**	others	*F. ferulaeoides* (Steud.) Korov	[61]
Ferulinkiol-1-hydroxy-5(2-methylbutyrate)	**11**	I	*F. linkii* Webb & Berthel.	[34]
Ferupennin A	**89**	II	*F. penninervis* Regel et Schmalh.	[44]
Ferupennin B	**90**	II	*F. penninervis* Regel et Schmalh.	[44]
Ferupennin C	**91**	II	*F. penninervis* Regel et Schmalh.	[44]
Ferupennin D	**92**	II	*F. penninervis* Regel et Schmalh.	[44]
Ferupennin E	**93**	II	*F. penninervis* Regel et Schmalh.	[44]
Ferupennin F	**94**	II	*F. penninervis* Regel et Schmalh.	[44]
Ferupennin G	**95**	II	*F. penninervis* Regel et Schmalh.	[44]
Ferupennin H	**96**	II	*F. penninervis* Regel et Schmalh.	[44]
Ferupennin I	**97**	II	*F. penninervis* Regel et Schmalh.	[44]
Ferupennin J	**98**	II	*F. penninervis* Regel et Schmalh.	[44]
Ferupennin K	**99**	II	*F. penninervis* Regel et Schmalh.	[44]
Ferupennin L	**100**	II	*F. penninervis* Regel et Schmalh.	[44]
Ferupennin L	**111**	II	*F. varia* (Schrenk) Trautv.	[35]
Ferupennin M	**101**	II	*F. penninervis* Regel et Schmalh.	[44]
Ferupennin N	**102**	II	*F. penninervis* Regel et Schmalh.	[44]
Ferupennin O	**112**	II	*F. penninervis* Regel et Schmalh.	[44]
Ferutidin	**14**	I	*F. elaeochytris* Korovin	[62]
			*lancerottensis* Parl.	[54]
			*F. licentiana* var. *tunshanica* (Su) Shan et Q.X.Liu	[57]
			*F. arrigonii* Bocchieri	[34]
			*F. communis* L.	[43]
			*F. communis* subsp. *communis*	[31]
			*F. glauca* subsp. *glauca*	[31,38]
			*F. kuhistanica* Korovin	[48]
Ferutinin	**18**	I	*F. hermonis* Boiss	[52]
			*F. jaeschkeana* Vatke	[59,60,63]
			*F. kuhistanica* Korovin	[64]
			*F. elaeochytris* Korovin	[62]
			*F. sinaica* L.	[37]
			*F. lancerottensis* Parl.	[54]
			*F. licentiana* var. *tunshanica* (Su) Shan et Q.X.Liu	[57]
			*F. kingdom-wardii* Wolff	[57]
			*F. communis* L.	[53,65]
Ferutionone	**33**	I	*F. jaeschkeana* Vatke	[63]
Fetidone A	**145**	others	*F. assa-foetida* L.	[66]
Fetidone B	**146**	others	*F. assa-foetida* L.	[66]
2*α*-Hydroxy ferutinin	**1**	I	*F. glauca* subsp. *glauca*	[31]
1*α*-Hydroxy-2-oxo-5*α*,7*β*-11*β*H-eudesm-3-en-6*α*,12-olide	**129**	IV	*F. penninervis* Regel et Schmalh.	[44]
2*β*-Hydroxy-3,4-epoxyjaes-chkeanadiol	**74**	I	*F. jaeschkeana* Vatke	[67]
14-(4′-Hydroxy-3′-methoxy-benzoyloxy)dauc-4,8-diene	**37**	I	*F. hermonis* Boiss	[52]
2*α*-Hydroxy-6*α*-*p*-methoxybenzoyl-10*β*-acetoxy-jaeschkeanadiol	**2**	I	*F. communis* subsp. *communis*	[31]
5-*p*-Hydroxybenzoyl ester of ferutiol	**55**	I	*F. sinaica* L.	[37]
14-(4′-Hydroxybenzoyloxy)-dauc-4,8-diene	**36**	I	*F. hermonis* Boiss	[52]
5*α*-*p*-Hydroxybenzoyloxydauc-2-ene-1-one	**23**	I	*F. kuhistanica* Korovin	[48]
6-*β*-*p*-Hydroxybenzoyloxygermacra-1(10),4-diene	**141**	V	*F. lycia* Boiss.	[22]
14-Hydroxy-dauc-4-ene	**68**	I	*F. sinaica* L.	[68]
(1*R*,4*R*)-4-Hydroxydauca-7-ene-6,9-dione	**64**	I	*F. hermonis* Boiss	[56]
(1*R*,4*R*)-4-Hydroxydauca-7-ene-6-one	**63**	I	*F. hermonis* Boiss	[56]
10*α*-Hydroxyfertidin	**30**	I	*F. arrigonii* Bocchieri	[43]
10-Hydroxylancerodiol-6-anisate	**59**	I	*F. vesceritensis* Coss. & Durieu ex Trab.	[45]
10-Hydroxylancerodiol-6-benzoate	**60**	I	*F. vesceritensis* Coss. & Durieu ex Trab.	[45]
14-Hydroxyvaginatin	**44**	I	*F. sinaica* L.	[37]
14-Hydroxyvaginatin	**86**	I	*F. sinaica* L.	[37]
Isolancerotriol	**72**	I	*F. sinaica* L.	[37,58]
5-Isovalerate of lapiferol	**83**	I	*F. communis* L.	[53]
			*F. linkii* Webb & Berthel.	[69]
Jaeschkeanadiol	**16**	I	*F. hermonis* Boiss	[52]
			*F. jaeschkeana* Vatke	[59,60,63]
			*F. kuhistanica* Korovin	[48,64]
			*F. elaeochytris* Korovin	[62]
			*F. sinaica* L.	[37]
			*F. communis* L.	[53]
			*F. lancerottensis* Parl.	[54]
Jaeschkeanadiol isovalerate	**12**	I	*F. linkii* Webb & Berthel.	[34]
Jaeskeanadiol angelate	**26**	I	*F. jaeschkeana* Vatke	[63]
			*F. lancerottensis* Parl.	[54]
			*F. elaeochytris* Korovin	[62]
Jaeskeanadiol salicylate	**27**	I	*F. elaeochytris* Korovin	[62]
Jaeskeanadiol veratrate	**29**	I	*F. arrigonii* Bocchieri	[43]
			*F. licentiana* var. *tunshanica* (Su) Shan et Q.X.Liu	[57]
Juniferdin	**121**	III	*F. lycia* Boiss.	[22]
Juniferin	**123**	III	*F. lycia* Boiss.	[22]
Juniferinin	**122**	III	*F. lycia* Boiss.	[22]
Kuhistaferone	**149**	others	*F. kuhistanica* Korovin	[70]
Kuhistanicaol A	**76**	I	*F. kuhistanica* Korovin	[48]
Kuhistanicaol D	**24**	I	*F. kuhistanica* Korovin	[48]
Kuhistanicaol E	**65**	I	*F. kuhistanica* Korovin	[48]
Kuhistanicaol F	**66**	I	*F. kuhistanica* Korovin	[48]
Kuhistanicaol G	**53**	I	*F. kuhistanica* Korovin	[48]
Kuhistanicaol H	**75**	I	*F. kuhistanica* Korovin	[64]
Kuhistanicaol I	**25**	I	*F. kuhistanica* Korovin	[64]
Kuhistanicaol J	**88**	I	*F. kuhistanica* Korovin	[64]
Lancerodiol-*p*-hydroxybenzoate	**58**	I	*F. lancerottensis* Parl.	[54]
			*F. jaeschkeana* Vatke	[67]
			*F. sinaica* L.	[58]
			*F. linkii* Webb & Berthel.	[71]
Lancerodiol-*p*-methoxybenzoate	**67**	I	*F. glauca* subsp. *glauca*	[31]
Lancerotriol-9-acetate-6-*p*-hydroaxybenzoate	**56**	I	*F. sinaica* L.	[46]
Lanerotriol-*p*-hydroxy-benzoate	**61**	I	*F. kuhistanica* Korovin	[48]
Lapidin	**35**	I	*F. lapidosa* Korov.	[72]
Lapidol	**34**	I	*F. jaeschkeana* Vatke	[67]
Lapidol isobutyrate	**9**	I	*F. linkii* Webb & Berthel.	[34]
Lapidol vanillate	**22**	I	*F. kuhistanica* Korovin	[48]
Lapidol-2-methybutyrate	**8**	I	*F. linkii* Webb & Berthel.	[34]
Lapidol-*p*-anisate	**10**	I	*F. linkii* Webb & Berthel.	[34]
Lapiferin	**77**	I	*F. vesceritensis* Coss. & Durieu ex Trab.	[73]
			*F. arrigonii* Bocchieri	[43]
Lasidiol-10-anisate	**43**	I	*F. vesceritensis* Coss. & Durieu ex Trab.	[45]
Lyciferin A	**116**	III	*F. lycia* Boiss.	[22]
Lyciferin B	**117**	III	*F. lycia* Boiss.	[22]
Lyciferin C	**118**	III	*F. lycia* Boiss.	[22]
Lyciferin D	**119**	III	*F. lycia* Boiss.	[22]
Lyciferin E	**120**	III	*F. lycia* Boiss.	[22]
l*α*,l0*β*-Epoxy-4-humden-6*β*-*p*-anisate	**127**	III	*F. linkii* Webb & Berthel.	[34]
l*α*,l0*β*-Epoxy-4-humden-6*β*-*p*-veratrate	**128**	III	*F. linkii* Webb & Berthel.	[34]
14-*p*-Methoxybenzoyl-4,5-epoxy-dauc-8-ene	**46**	I	*F. communis* subsp. *communis*	[31]
Nerolidol	**144**	others	*F. fukanensis* K. M. Shen	[74]
Oopodin	**139**	IV	*F. varia* (Schrenk) Trautv.	[35]
2-Oxoferutidin	**32**	I	*F. arrigonii* Bocchieri	[43]
Pallinin or 6*α*,10*α*-diangeloyl-jaeschkeanadiol	**7**	I	*F. communis* subsp. *communis*	[31]
Penninnervin	**131**	IV	*F. penninervis* Regel et Schmalh.	[44]
*p*-Hydroxybenzoylfervanol	**125**	III	*F. lycia* Boiss.	[22]
Spathulenol	**115**	II	*F. varia* (Schrenk) Trautv.	[35]
Teferidin	**17**	I	*F. hermonis* Boiss	[52]
			*F. elaeochytris* Korovin	[62]
			*F. jaeschkeana* Vatke	[63]
			*F. sinaica* L.	[37]
Teferin	**21**	I	*F. hermonis* Boiss	[52]
			*F. jaeschkeana* Vatke	[59,60,63]
			*F. kuhistanica* Korovin	[48,64]
			*F. kingdom-ardii* Wolff	[57]
			*F. elaeochytris* Korovin	[62]
4*β*, 8*β*, 9*α*-Trihydroxy-6*α*-*p*-hydroxybenzoyoxydaucane	**87**	I	*F. sinaica* L.	[37,58]
Tunetanin A	**15**	I	*F. tunetana* Pom	[75]
Vaginatin	**40**	I	*F. communis* subsp. *communis*	[40]
5*α*-Vanillate-2,3-epoxy-jaescheanadiol	**79**	I	*F. kuhistanica* Korovin	[48]
			*F. jaeschkeana* Vatke	[67]
Vanilloylfervanol	**126**	III	*F. lycia* Boiss.	[22]
6-*β*-Vanilloyloxygermacra-1(10),4-diene	**142**	V	*F. lycia* Boiss.	[22]
Vesceritenone	**85**	I	*F. vesceritensis* Coss. & Durieu ex Trab.	[45]
Webbiol angelate	**69**	I	*F. linkii* Webb & Berthel.	[71]
-	**110**	II	*F. varia* (Schrenk) Trautv.	[35]
-	**133**	IV	*F. varia* (Schrenk) Trautv.	[35]
-	**134**	IV	*F. varia* (Schrenk) Trautv.	[35]
-	**135**	IV	*F. varia* (Schrenk) Trautv.	[35]
-	**136**	IV	*F. varia* (Schrenk) Trautv.	[35]
-	**137**	IV	*F. varia* (Schrenk) Trautv.	[35]

**Table 2:** Sesquiterpene coumarins in *Ferula* plants.

Names	No.	Types	Species	References
*ε*-Acetoxy,4-acetylferulenol	**357**	IIIa	*F. communis* var*. genuina*	[76]
10′*R*-Acetoxy-11′-hydroxyumbelliprenin	**167**	Ia	*F. assa-foetida* L.	[10]
5′-Acetoxy-8′-hydroxyumbelliprenin	**156**	Ia	*F. assa*-*foetida* L.	[10]
8′-Acetoxy-5′-hydroxyumbelliprenin	**155**	Ia	*F. assa*-*foetida* L.	[77]
(*Z*)-*ω*-Acetoxyferprenin	**401**	IIIc	*F. communis* L.	[78,79]
(*E*)-*ω*-Acetoxyferprenin	**402**	IIIc	*F. communis* L.	[78,79]
(*E*)-*ω*-Acetoxyferulenol	**352**	IIIa	*F. communis* L.	[80]
(*Z*)-*ω*-Acetoxyferulenol	**353**	IIIa	*F. communis* L.	[80]
8-*O*-Acetyl-sinkiangenorin F	**209**	Ib	*F. sinkiangensis* K. M. Shen	[81]
Ammoresinal	**356**	IIIa	*F. vesceritensis* Coss. & Durieu ex Trab.	[45]
3-Angeloxycoladin	**242**	Ic	*F. vesceritensis* Coss. & Durieu ex Trab.	[5]
Asacoumarin A	**154**	Ia	*F. assa-foetida* L.	[82]
			*F. foetida* (Bunge) Regel	[83]
Asacoumarin B	**217**	Ib	*F. assa-foetida* L.	[82]
Asimafoetida	**189**	Ib	*F. assafoetida* Linn.	[84]
Asimafoetidnol	**203**	Ib	*F. assa-foetida* L.	[85]
Assafoetidin	**190**	Ib	*F. fukanensis* K. M. Shen	[86]
			*F. lehmannii* Boss.	[87]
			*F. assafoetida* Linn.	[88]
Assafoetidnol A	**230**	Ic	*F. assa-foetida* L.	[89]
Assafoetidnol B	**231**	Ic	*F. assa-foetida* L.	[89]
Badrakemin	**238**	Ic	*F. teterrima* Kar. et Kir.	[90]
			*F. badrakema* Kos.-Pol.	[91]
Badrakemin acetate	**239**	Ic	*F. teterrima* Kar. et Kir.	[90]
			*F. badrakema* Kos.-Pol.	[41]
Badrakemone	**237**	Ic	*F. fukanensis* K. M. Shen	[86]
			*F. teterrima* Kar. et Kir.	[90]
			*F. persica* Willd	[92]
			*F. nevskii* Korov.	[93]
Cauferidin	**245**	Ic	*F. conocaula* Korov.	[94]
Cauferin	**249**	Ic	*F. conocaula* Korov.	[94]
Cauferinin	**259**	Ic	*F. conocaula* korov.	[95]
			*F. samarkandica* Korovin	[96]
Cauferoside	**235**	Ic	*F. gumosa* Boiss.	[97]
			*F. conocaula* Korov.	[98]
Cauloside	**252**	Ic	*F. conocaula* Korov.	[99]
Cocanicin	**160**	Ia	*F. cocanica*	[99]
Coladin	**232**	Ic	*F. sinkiangensis* K. M. Shen	[5]
			*F. tunetana* Pom	[5,75]
			*F. campestris* (Besser) Grecescu	[100]
Colladonin	**240**	Ic	*F. teterrima* Kar. et Kir.	[90]
			*F. foetida* (Bunge) Regel	[83]
			*F. sinkiangensis* K. M. Shen	[101]
			*F. sinaica* L.	[5]
			*F. tunetana* POM	[75]
			*F. campestris* (Besser) Grecescu	[100]
Colladonin isovalerate	**241**	Ic	*F. loscossi* (Lge) Wk	[102]
Communiferulin A	**366**	IIIb	*F. communis* L.	[103]
Communiferulin B	**367**	IIIb	*F. communis* L.	[103]
Communiferulin C	**368**	IIIb	*F. communis* L.	[103]
Conferdione	**283**	Ic	*F. flabelliloba* Rech. f. & Aell	[104,105]
Conferin	**286**	Ic	*F. conocaula* Korov.	[106]
Conferol	**267**	Ic	*F. assa-foetida* L.	[10]
			*F. gumosa* Boiss.	[97]
			*F. conocaula* Korov.	[107]
Conferol acetate	**264**	Ic	*F. badrakema* Kos.-Pol.	[41]
Conferone	**265**	Ic	*F. flabelliloba* Rech. f. & Aell	[104]
			*F. badrakema* Kos.-Pol.	[41,108]
Conferoside	**236**	Ic	*F. gumosa* Boiss.	[97]
			*F. conocaula* Korov.	[98]
Conferoside	**285**	Ic	*F. conocaula* Korov.	[98]
Deacetylkellerin	**323**	Ic	*F. kelleri* K.-Pol.	[109]
			*F. kokanica* Regel & Schmalh.	[110]
Deacetyltadshikorin	**158**	Ia	*F. tadshikorum* M. Pimen	[111]
Diastereomer-samarcandin	**289**	Ic	*F. sinaica* L.	[112]
2,3-Dihydro-7-hydroxy-2R*,3R*-dimethyl-2-[4,8-dimethyl-3(*E*),7-nonadien-6-onyl]-furo[3,2-*c*]coumarin	**399**	IIIb	*F. fukanensis* K. M. Shen	[113]
2,3-Dihydro-7-hydroxy-2R*,3R*-dimethyl-2-[4,8-dimethyl-3(*E*),7-nonadienyl]-furo[3,2-*c*]coumarin	**386**	IIIb	*F. ferulaeoides* (Steud.) Korov	[114]
2,3-Dihydro-7-hydroxy-2R*,3R*-dimethyl-2-[4-methyl-5-(4-methyl-2-furyl)-3(*E*)-pentenyl]-furo[3,2-*c*]coumarin	**394**	IIIb	*F. ferulaeoides* (Steud.) Korov	[114]
2,3-Dihydro-7-hydroxy-2S*,3R*-dimethyl-2-[4,8-dimethyl-3(*E*),7-nonadien-6-onyl]-furo[3,2-*c*]coumarin	**390**	IIIb	*F. ferulaeoides* (Steud.) Korov	[114]
2,3-Dihydro-7-hydroxy-2S*,3R*-dimethyl-2-[4,8-dimethyl-3(*E*),7-nonadienyl]-furo[3,2-*c*]coumarin	**383**	IIIb	*F. ferulaeoides* (Steud.) Korov	[114]
2,3-Dihydro-7-hydroxy-2S*,3R*-dimethyl-2-[4-methyl-5-(4-methyl-2-furyl)-3(*E*)-pentenyl]-furo[3,2-*c*]coumarin	**392**	IIIb	*F. ferulaeoides* (Steud.) Korov	[114]
2,3-Dihydro-7-hydroxy-2S*,3R*-dimethyl-3-[4,8-dimethyl-3(*E*),7-nonadienyl]-furo[3,2-*c*]coumarin	**370**	IIIb	*F. ferulaeoides* (Steud.) Korov	[114]
2,3-Dihydro-7-hydroxy-2S*,3R*-dimethyl-3-[4-methyl-5-(4-methyl-2-furyl)-3(*E*)-pentenyl]-furo[3,2-*c*]coumarin	**373**	IIIb	*F. ferulaeoides* (Steud.) Korov	[114]
2,3-Dihydro-7-hydroxy-2S*,3S*-dimethyl-2-[4,8-dimethyl-3(E),7-nonadienyl]-furo[3,2-c]coumarin	**389**	IIIb	*F. ferulaeoides* (Steud.) Korov	[115]
2,3-Dihydro-7-hydroxy-2S*,3S*-dimethyl-3-[4,8-dimethyl-3(*E*),7-nonadienyl]-furo[3,2-*c*]coumarin	**372**	IIIb	*F. ferulaeoides* (Steud.) Korov	[114]
(2S*,3R*)-2,3-Dihydro-7-hydroxy-2-[(3*E*)-8-hydroxy-4,8-dimethylnon-3-en-1-yl]-2,3-dimethyl-4H-furo[3,2-*c*][1]benzopyran-4-one	**364**	IIIb	*F. ferulaeoides* (Steud.) Korov	[116]
(2S*,3R*)-2,3-Dihydro-2-[(3*E*)-8-hydroxy-4,8-dimethylnon-3-en-1-yl]-7-methoxy-2,3-dimethyl-4H-furo[3,2-*c*][1]benzopyran-4-one	**365**	IIIb	*F. ferulaeoides* (Steud.) Korov	[116]
2,3-Dihydro-7-methoxy-2R*,3R*-dimethyl-2-[4,8-dimethyl-3(E),7-nonadienyl]-furo[3,2-c]coumarin	**387**	IIIb	*F. ferulaeoides* (Steud.) Korov	[114]
2,3-Dihydro-7-methoxy-2S*,3R*-dimethyl-2-[4,8-dimethyl-3(*E*),7-nonadien-6-onyl]-furo[3,2-*c*]coumarin	**391**	IIIb	*F. ferulaeoides* (Steud.) Korov	[114]
2,3-Dihydro-7-methoxy-2S*,3R*-dimethyl-2-[4,8-dimethyl-3(*E*),7-nonadienyl]-furo[3,2-*c*]coumarin	**384**	IIIb	*F. ferulaeoides* (Steud.) Korov	[114]
2,3-Dihydro-7-methoxy-2S*,3R*-dimethyl-2-[4-methyl-5-(4-methyl-2-furyl)-3(*E*)-pentenyl]-furo[3,2-*c*]coumarin	**393**	IIIb	*F. ferulaeoides* (Steud.) Korov	[114]
2,3-Dihydro-7-methoxy-2S*,3R*-dimethyl-3-[4,8-dimethyl-3(*E*),7-nonadienyl]-furo[3,2-*c*]coumarin	**371**	IIIb	*F. ferulaeoides* (Steud.) Korov	[114]
6′,7′-Dihydroxy-karatavicinol	**169**	Ia	*F. sinaica* L.	[37]
(2S*,3S*)-3-[(3*E*)-4,8-Dimethylnona-3,7-dien-1-yl]-2,3-dihydro-7-methoxy-2,3-dimethyl-4H-furo[3,2-*c*][1]benzopyran-4-one	**363**	IIIb	*F. ferulaeoides* (Steud.) Korov	[116]
Drimatol B	**345**	Ic	*F. jaeschkeana* Vatke	[63]
*Ent*-Colladonin	**248**	Ic	*F. sinkiangensis* K. M. Shen	[117]
*Epi*-Conferdione	**272**	Ic	*F. assa-foetida* L.	[10]
			*F. foetida* (Bunge) Regel	[83]
Episamarcandin	**305**	Ic	*F. sinkiangensis* K. M. Shen	[118]
			*F. sinaica* L.	[119]
Episamarcandin acetate	**295**	Ic	*F. assa-foetida* L.	[120]
Epoxyfarnochrol	**175**	Ia	*F. jaeschkeana* Vatke	[63]
Ethyl galbanate	**215**	Ib	*F. pseudalliacea* Rech.f.	[121]
Farnesiferol A	**228**	Ic	*F. assafoetida* Linn.	[84]
			*F. assa-foetida* L.	[10]
			*F. vesceritensis* Coss. & Durieu ex Trab.	[45]
			*F. persica* Willd	[92]
Farnesiferol B	**186**	Ib	*F. sinkiangensis* K. M. Shen	[118]
			*F. szowitsiana* DC.	[122]
			*F. flabelliloba* Rech. f. & Aell	[104]
			*F. lehmannii* Boss.	[123]
			*F. asafoetida* L.	[124]
			*F. assa*-*foetida* L.	[10]
			*F. persica* Willd	[92]
Farnesiferol C	**202**	Ib	*F. lehmannii* Boss.	[87]
			*F. sinkiangensis* K. M. Shen	[118]
			*F. szowitsiana* DC.	[122,125]
			*F. asafoetida* L.	[124]
			*F. assafoetida* Linn.	[84]
Farnesiferone B	**185**	Ib	*F. flabelliloba* Rech. f. & Aell	[104]
Fecarpin	**330**	Ic	*F. microcarpa* Korovin	[126]
Fekolin	**193**	Ib	*F. kopetdagensis* Eug. Kor.	[99,127]
Fekolone	**187**	Ib	*F. fukanensis* K. M. Shen	[86]
			*F. sinkiangensis* K. M. Shen	[118,128]
			*F. kopetdagensis* Eug. Kor.	[127]
Fekolone	**195**	Ib	*F. kopetdagensis* Eug. Kor.	[99,127]
Fekrol	**196**	Ib	*F. krylovii* Korov.	[129]
Fekrynol	**211**	Ib	*F. sinkiangensis* K. M. Shen	[118]
			*F. lehmannii* Boss.	[87]
			*F. krylovii* Korov.	[130]
Fekrynol acetate	**212**	Ib	*F. lehmannii* Boss.	[123]
			*F. krylovii* Korov.	[130]
Fepaldlin	**322**	Ic	*F. pallida* Korovin	[99]
Fercoprenol	**369**	IIIb	*F. communis* subsp*. communis*	[131]
Ferocaulicin	**288**	Ic	*F. conocaula* Korov.	[132]
Ferocaulidin	**287**	Ic	*F. gumosa* Boiss.	[97]
			*F. badrakema* Kos.-Pol	[41,108]
			*F. conocaula* Korov.	[132]
Ferocaulin	**282**	Ic	*F. conocaula* Korov.	[132]
Ferocaulinin	**284**	Ic	*F. conocaula* Korov.	[132]
Feropolidin	**263**	Ic	*F. polyantha* Korovin	[133,134]
			*F. vicaria* Korovin	[135]
Feropolin	**207**	Ib	*F. polyantha* Korovin	[133,134]
Feropolol	**204**	Ib	*F. polyantha* Korovin	[133,134]
			*F. vicaria* Korovin	[135]
Feropolone	**206**	Ib	*F. polyantha* Korovin	[133,134]
			*F. vicaria* Korovin	[135]
Feroside	**164**	Ia	*F. korshinskyi* Eug. Korov	[136]
Ferprenin	**405**	IIIc	*F. communis* L.	[78,79]
Ferubungeanol a	**307**	Ic	*F. bungeana* Kitag.	[137]
Ferubungeanol b	**308**	Ic	*F. bungeana* Kitag.	[137]
Ferubungeanol c	**309**	Ic	*F. bungeana* Kitag.	[137]
Ferubungeanol d	**310**	Ic	*F. bungeana* Kitag.	[137]
Ferubungeanol e	**311**	Ic	*F. bungeana* Kitag.	[137]
Ferubungeanol f	**312**	Ic	*F. bungeana* Kitag.	[137]
Ferubungeanol g	**313**	Ic	*F. bungeana* Kitag.	[137]
Ferubungeanol h	**314**	Ic	*F. bungeana* Kitag.	[137]
Ferucrin isobutyrate	**324**	Ic	*F. foetidissima* Regel & Schmalh.	[138]
Ferucrinone	**325**	Ic	*F. foetidissima* Regel & Schmalh.	[138]
Ferukrin	**319**	Ic	*F. kopetdagensis* Eug. Kor.	[139]
			*F. krylovii* Korov.	[140]
Ferukrin acetate	**320**	Ic	*F. kopetdagensis* Eug. Kor.	[139]
Ferulenol	**349**	IIIa	*F. communis* var*. genuina*	[141,142]
Ferulenoloxy ferulenol	**361**	IIIa	*F. communis* var. *genuina*	[76]
Ferulin A	**395**	IIIb	*F. ferulaeoides* (Steud.) Korov	[143]
Ferulin B	**374**	IIIb	*F. ferulaeoides* (Steud.) Korov	[143]
Ferulin C	**375**	IIIb	*F. ferulaeoides* (Steud.) Korov	[143]
Ferulsinaic acid	**218**	Ib	*F. sinaica* L.	[144,145]
Ferusingensine A	**171**	Ia	*F. sinkiangensis* K. M. Shen	[146]
Ferusingensine B	**172**	Ia	*F. sinkiangensis* K. M. Shen	[146]
Ferusingensine C	**173**	Ia	*F. sinkiangensis* K. M. Shen	[146]
Ferusingensine D	**174**	Ia	*F. sinkiangensis* K. M. Shen	[146]
Ferusingensine E	**168**	Ia	*F. sinkiangensis* K. M. Shen	[146]
Ferusingensine F	**183**	Ib	*F. sinkiangensis* K. M. Shen	[146]
Ferusingensine G	**177**	Ib	*F. sinkiangensis* K. M. Shen	[146]
Ferusingensine H	**326**	Ic	*F. sinkiangensis* K. M. Shen	[146]
Ferusinol	**338**	Ic	*F. sinaica* L.	[112]
Feselol	**268**	Ic	*F. assa-foetida* L.	[10]
			*F. flabelliloba* Rech. f. & Aell	[104]
			*F. badrakema* Kos.-Pol	[41,108]
			*F. vesceritensis* Coss. & Durieu ex Trab.	[45]
Feselol angelate	**270**	Ic	*F. diversivittata* Regel & Schmalh.-Rech.	[147]
Feshurin	**301**	Ic	*F. teterrima* Kar. et Kir.	[90,148]
Feshurin acetate	**302**	Ic	*F. teterrima* Kar. et Kir.	[90]
Fesinkin A	**184**	Ib	*F. sinkiangensis* K. M. Shen	[149]
Fesinkin B	**178**	Ib	*F. sinkiangensis* K. M. Shen	[149]
Fesinkin C	**179**	Ib	*F. sinkiangensis* K. M. Shen	[149]
4′*E*-Fesinkin D	**180**	Ib	*F. sinkiangensis* K. M. Shen	[149]
4′*Z*-Fesinkin D	**181**	Ib	*F. sinkiangensis* K. M. Shen	[149]
Fesinkin E	**339**	Ic	*F. sinkiangensis* K. M. Shen	[149]
Fesinkin F	**340**	Ic	*F. sinkiangensis* K. M. Shen	[149]
Fesinkin G	**341**	Ic	*F. sinkiangensis* K. M. Shen	[149]
Feterin	**250**	Ic	*F. teterrima* Kar. et Kir.	[150]
Feterin acetate	**251**	Ic	*F. teterrima* Kar. et Kir.	[150]
Flabellilobin A	**188**	Ib	*F. flabelliloba* Rech. f. & Aell	[104]
Flabellilobin B	**192**	Ib	*F. flabelliloba* Rech. f. & Aell	[104]
Fnarthexol	**279**	Ic	*F. narthex* Boiss	[151]
Fnarthexone	**246**	Ic	*F. narthex* Boiss	[151]
Foetidin	**346**	II	*F. assa-foetida* L.	[152]
Foetidin acetate	**347**	II	*F. marmarica* Asch. & Taub.	[153]
Foetidone	**348**	II	*F. marmarica* Asch. & Taub.	[153]
Foliferin	**205**	Ib	*F. folioca* Lipsky	[154]
			*F. schtschurowskiana* Regel & Schmalh.	[99]
Fukanefuromarin A	**376**	IIIb	*F. fukanensis* K. M. Shen	[113]
Fukanefuromarin B	**377**	IIIb	*F. fukanensis* K. M. Shen	[113]
Fukanefuromarin C	**378**	IIIb	*F. fukanensis* K. M. Shen	[113]
Fukanefuromarin D	**379**	IIIb	*F. fukanensis* K. M. Shen	[113]
Fukanefuromarin E	**380**	IIIb	*F. fukanensis* K. M. Shen	[155]
Fukanefuromarin F	**381**	IIIb	*F. fukanensis* K. M. Shen	[155]
Fukanefuromarin G	**382**	IIIb	*F. fukanensis* K. M. Shen	[155]
Fukanefuromarin H	**385**	IIIb	*F. fukanensis* K. M. Shen	[156]
Fukanefuromarin I	**388**	IIIb	*F. fukanensis* K. M. Shen	[156]
Fukanefuromarin J	**396**	IIIb	*F. fukanensis* K. M. Shen	[156]
Fukanefuromarin K	**397**	IIIb	*F. fukanensis* K. M. Shen	[156]
Fukanefuromarin L	**398**	IIIb	*F. fukanensis* K. M. Shen	[156]
Fukanefuromarin M	**407**	IIIc	*F. fukanensis* K. M. Shen	[156]
Fukanemarin A	**358**	IIIa	*F. fukanensis* K. M. Shen	[113]
Fukanemarin B	**360**	IIIa	*F. fukanensis* K. M. Shen	[155]
Galbanic acid	**213**	Ib	*F. szowitsiana* DC.	[122,157]
			*F. asafoetida* L.	[124]
			*F. assa-foetida* L.	[10]
Gummosin	**225**	Ic	*F. persica* Willd	[92]
Gumoside A	**233**	Ic	*F. gumosa* Boiss.	[97]
Gumoside B	**234**	Ic	*F. gumosa* Boiss.	[97]
Gumosin	**315**	Ic	*F. gumosa* Boiss.	[97]
4′-Hydroxy kamolonol acetate	**277**	Ic	*F. pseudalliacea* Rech.f.	[158]
(*Z*)-*ω*-Hydroxyferprenin	**403**	IIIc	*F. communis* L.	[78,79]
(*E*)-*ω*-Hydroxyferprenin	**404**	IIIc	*F. communis* L.	[78,79]
(*E*)-*ω*-Hydroxyferulenol	**350**	IIIa	*F. communis* L.	[80]
(*Z*)-*ω*-Hydroxyferulenol	**351**	IIIa	*F. communis* L.	[80]
*ε*-Hydroxyferulenol	**355**	IIIa	*F. communis* L.	[76]
13-Hydroxyfeselol	**271**	Ic	*F. vesceritensis* Coss. & Durieu ex Trab.	[5]
			*F. tunetana* POM	[75]
5′-Hydroxyumbelliprenin	**151**	Ia	*F. assa-foetida* L.	[77]
			*F. assa*-*foetida* L.	[10]
8′-Hydroxyumbelliprenin	**152**	Ia	*F. assa*-*foetida* L.	[77]
Isoferprenin	**406**	IIIc	*F. communis* var. *genuina*	[159]
Isofeterin	**243**	Ic	*F. teterrima* Kar. et Kir.	[160]
Isosamarcandin	**303**	Ic	*F. sinaica* L.	[68]
			*F. sinkiangensis* K. M. Shen	[118]
			*F. microloba* Boiss.	[161]
Isosamarkandin	**293**	Ic	*F. sinkiangensis* K. M. Shen	[117]
Isosamarkandin angelate	**304**	Ic	*F. arrigonii* Bocchieri	[43]
Isosmarcandin	**297**	Ic	*F. tunetana* POM	[75]
Kamolol	**331**	Ic	*F. penninervis* Regel et Schmalh.	[162]
(3′*S*, 4′*S*, 5′*R*, 8′*S*, 9′*S*, 10′*S*)-Kamolol acetate	**327**	Ic	*F. sinkiangensis* K. M. Shen	[146]
Kamolone	**332**	Ic	*F. penninervis* Regel et Schmalh.	[162]
Kamolonol	**334**	Ic	*F. assa-foetida* L.	[10]
			*F. pseudalliacea* Rech.f.	[158]
Kamolonol acetate	**299**	Ic	*F. pseudooreoselinum* Koso-Pol.	[121]
Kamonolol acetate	**278**	Ic	*F. pseudalliacea* Rech.f.	[163]
Karatavic acid	**216**	Ib	*F. karatavica* Regel & Schmalh.	[164,165,166]
Karatavicin	**163**	Ia	*F. karatavica* Regel & Schmalh.	[148]
Karatavicinol	**161**	Ia	*F. foetida* (Bunge) Regel	[83]
			*F. asafoetida* L.	[124]
			*F. assa-foetida* L.	[10]
			*F. karatavica* Rgl. et Schmalh.	[167]
Kellerin	**321**	Ic	*F. kelleri* K.-Pol.	[109]
			*F. kokanica* Regel & Schmalh.	[110]
Kokanidin	**306**	Ic	*F. kokanica* Regel & Schmalh.	[110]
Kopeolin	**197**	Ib	*F. gummosa* Boiss.	[168]
			*F. kopetdagensis* Eug. Kor.	[169,170]
Kopeolone	**199**	Ib	*F. kopetdagensis* Eug. Kor.	[169]
Kopeoside	**198**	Ib	*F. gummosa* Boiss.	[168]
			*F. kopetdagensis* Eug. Kor.	[169,170]
Kopetdaghin	**194**	Ib	*F. gummosa* Boiss.	[168]
			*F. kopetdagensis* Eug. Kor.	[169,171]
Lehmannolol	**300**	Ic	*F. sinkiangensis* K. M. Shen	[160]
			*F. assa-foetida* L.	[10]
Lehmannolone	**298**	Ic	*F. fukanensis* K. M. Shen	[86]
			*F. sinkiangensis* K. M. Shen	[160]
			*F. lehmannii* Boiss.	[172]
Lehmannolone A	**200**	Ib	*F. lehmannii* Boss.	[87]
Lehmferidin	**244**	Ic	*F. lehmannii* Boss.	[173]
Lehmferin	**191**	Ib	*F. flabelliloba* Rech. f. & Aell	[104]
			*F. assa-foetida* L.	[10]
			*F. lehmannii* Boss.	[173]
Ligupersin A	**273**	Ic	*F. assa-foetida* L.	[10]
			*F. flabelliloba* Rech. f. & Aell	[104]
			*F. gumosa* Boiss.	[97]
			*F. badrakema* Kos.-Pol.	[108]
Methyl galbanate	**214**	Ib	*F. szowitsiana* DC.	[122]
			*F. assa-foetida* L.	[10]
			*F. microloba* Boiss.	[161]
Microlobidene	**335**	Ic	*F. microloba* Boiss.	[174]
Microlobin	**333**	Ic	*F. assa-foetida* L.	[10,161]
Mogoltacin	**266**	Ic	*F. badrakema* Kos.-Pol.	[41,108]
Mogoltadone	**227**	Ic	*F. mogoltavica* Lipsky ex Korovin	[175]
Mogoltavicin	**329**	Ic	*F. mogoltavica* Lipsky ex Korovin	[176]
Mogoltavidin	**328**	Ic	*F. mogoltavica* Lipsky ex Korovin	[176]
Mogoltavin	**280**	Ic	*F. mogoltavica* Lipsky ex Korovin	[177]
Mogoltavinin	**281**	Ic	*F. mogoltavica* Lipsky ex Korovin	[177]
Moschatyl acetate	**269**	Ic	*F. incisoserrata* Pimenov & J.V.Baranova	[178]
Nevskone	**296**	Ic	*F. neveskii* Korovin	[99]
(*E*)-*ω*-Oxoferprenin	**400**	IIIc	*F. communis* L.	[78,79]
(*E*)-*ω*-Oxoferulenol	**354**	IIIa	*F. communis* L.	[80]
Pallidone A	**359**	IIIa	*F. pallida* Korovin	[179]
Pallidone B	**362**	IIIb	*F. pallida* Korovin	[179]
Persicaoside A	**342**	Ic	*F. persica* Willd	[180]
Persicaoside B	**247**	Ic	*F. persica* Willd	[180]
Persicaoside C	**165**	Ia	*F. persica* Willd	[180]
Persicaoside D	**166**	Ia	*F. persica* Willd	[180]
Polyanthin	**229**	Ic	*F. assa-foetida* L.	[10]
			*F. polyanthum* Eug. Korov.	[181]
Polyanthinin	**226**	Ic	*F. polyantha* Korovin	[99]
			*F. polyanthum* Eug. Korov.	[181]
(8′*S*,9′*S*,10′*S*)-Propionyl-fekrynol	**182**	Ib	*F. sinkiangensis* K. M. Shen	[146]
Reoselin	**162**	Ia	*F. kirialovii* Pimenov	[182]
			*F. korshinskyi* Eug. Korov	[136]
			*F. pseudooreoselinum* Koso-Pol.	[183,184]
Samarcandicin A	**253**	Ic	*F. samarkandica* Korovin	[96]
Samarcandicin B	**254**	Ic	*F. samarkandica* Korovin	[96]
Samarcandicin C	**255**	Ic	*F. samarkandica* Korovin	[96]
Samarcandicin D	**256**	Ic	*F. samarkandica* Korovin	[96]
Samarcandicin E	**223**	Ic	*F. samarkandica* Korovin	[96]
Samarcandicin F	**257**	Ic	*F. samarkandica* Korovin	[96]
Samarcandicin G	**224**	Ic	*F. samarkandica* Korovin	[96]
Samarcandicin H	**258**	Ic	*F. samarkandica* Korovin	[96]
Samarcandin	**291**	Ic	*F. samarcandica* kor.	[185]
			*F. teterrima* Kar. et Kir.	[117]
			*F. huber-morathii* Peşan	[246]
Samarcandin acetate	**290**	Ic	*F. teterrima* Kar. et Kir.	[117]
			*F. pseudooreoselinum* Koso-Pol.	[186]
			*F. huber-morathii* Peşan	[246]
Samarcandone	**292**	Ic	*F. samarcandica* kor.	[185]
			*F. sinaica* L.	[5]
			*F. huber-morathii* Peşan	[246]
Saradaferin	**261**	Ic	*F. assafoetida* Linn.	[187]
Seravschanin A	**274**	Ic	*F. seravschanica* Pimenov & J.V.Baranova	[188]
Seravschanin B	**275**	Ic	*F. seravschanica* Pimenov & J.V.Baranova	[188]
Seravschanin C	**276**	Ic	*F. seravschanica* Pimenov & J.V.Baranova	[188]
Seravschanin D	**170**	Ia	*F. seravschanica* Pimenov & J.V.Baranova	[188]
Seravschanin E	**157**	Ia	*F. seravschanica* Pimenov & J.V.Baranova	[188]
Sinkiangenol A	**220**	Ic	*F. sinkiangensis* K. M. Shen	[189]
Sinkiangenol B	**221**	Ic	*F. sinkiangensis* K. M. Shen	[189]
Sinkiangenol C	**176**	Ib	*F. sinkiangensis* K. M. Shen	[189]
Sinkiangenol D	**294**	Ic	*F. sinkiangensis* K. M. Shen	[189]
Sinkiangenol E	**222**	Ic	*F. sinkiangensis* K. M. Shen	[189]
Sinkiangenorin D	**219**	Ib	*F. sinkiangensis* K. M. Shen	[128]
Sinkiangenorin E	**344**	Ic	*F. sinkiangensis* K. M. Shen	[30]
Sinkiangenorin F	**208**	Ib	*F. sinkiangensis* K. M. Shen	[81]
(3′*S*,8′*R*,9′*S*,10′*R*)-Sinkianol A	**343**	Ic	*F. sinkiangensis* K. M. Shen	[190]
(3′*R*,5′*R*,10′*R*)-Sinkianol B	**210**	Ib	*F. sinkiangensis* K. M. Shen	[190]
Sinkianone	**201**	Ib	*F. sinkiangensis* K. M. Shen	[160]
			*F. lehmannii* Boss.	[87]
Sumferin	**262**	Ic	*F. sumbul* Hook	[191]
Szowitsiacoumarin A	**316**	Ic	*F. szowitsiana* DC.	[122]
Szowitsiacoumarin B	**317**	Ic	*F. szowitsiana* DC.	[122]
Tadzhiferin	**153**	Ia	*F. assa*-*foetida* L.	[77]
			*F. tadshikorum* M. Pimen	[192]
Tadzhikorin	**159**	Ia	*F. tadshikorum* M. Pimen	[192]
Tavicone	**337**	Ic	*F. aitchisonii* K.-Pol.	[166]
Tunetacoumarin A	**318**	Ic	*F. tunetana* POM	[75]
Umbelliprenin	**150**	Ia	*F. aitchisonii* K.-Pol.	[166]
			*F. arrigonii* Bocchieri	[43]
			*F. assafoetida* Linn.	[77,124]
			*F. campestris* (Besser) Grecescu	[100]
			*F. fukanensis* K. M. Shen	[86]
			*F. sinkiangensis* K. M. Shen	[193]
			*F. flabelliloba* Rech. f. & Aell	[104]
			*F. tunetana* POM	[75]
			*F. persica* Willd	[33]
			*F. szowitsiana* DC.	[122]
-	**260**	Ic	*F. sinaica* L.	[194]
-	**336**	Ic	*F. sinaica* L.	[68]

**Table 3:** Sesquiterpene chromones in *Ferula* plants.

Names	No.	Species	References
2,3-Dihydro-7-hydroxy-2S*,3R*-dimethyl-2-[4,8-dimethyl-3(*E*),7-nonadienyl]-furo[2,3-*b*]chromone	**408**	*F. ferulaeoides* (Steud.) Korov	[115]
2,3-Dihydro-7-hydroxy-2S*,3R*-dimethyl-2-[4-methyl-5-(4-methyl-2-furyl)-3(*E*),7-pentenyl]-furo[2,3-*b*]chromone	**409**	*F. ferulaeoides* (Steud.) Korov	[115]
2,3-Dihydro-7-hydroxy-2R*,3R*-dimethyl-2-[4-methyl-5-(4-methyl-2-furyl)-3(*E*),7-pentenyl]-furo[2,3-*b*]chromone	**410**	*F. ferulaeoides* (Steud.) Korov	[115]
Ferchromone	**420**	*F. communis* subsp. *communis*	[131]
Ferchromonol	**421**	*F. communis* subsp. *communis*	[131]
(+)-Ferulasin	**422**	*F. sinkiangensis* K. M. Shen	[195]
(-)-Ferulasin	**423**	*F. sinkiangensis* K. M. Shen	[195]
Ferulin D	**418**	*F. ferulaeoides* (Steud.) Korov	[143]
Ferulin E	**419**	*F. ferulaeoides* (Steud.) Korov	[143]
Fukanefurochromone A	**411**	*F. fukanensis* K. M. Shen	[196]
Fukanefurochromone B	**412**	*F. fukanensis* K. M. Shen	[196]
Fukanefurochromone C	**413**	*F. fukanensis* K. M. Shen	[196]
Fukanefurochromone D	**414**	*F. fukanensis* K. M. Shen	[196]
Fukanefurochromone E	**415**	*F. fukanensis* K. M. Shen	[196]
Pallidone I	**416**	*F. pallida* Korovin	[197]
Pallidone J	**417**	*F. pallida* Korovin	[197]

**Table 4:** Sesquiterpene phenylpropanoids in *Ferula* plants.

Names	No.	Species	References
3-(2,4-dihydroxybenzoyl)-4R*,5R*-dimethyl-5-[4,8-dimethyl-3(*E*),7(*E*)-nonadien-1-yl]tetra-hydro-2-furanone	**443**	*F. ferulaeoides* (Steud.) Korov.	[198,199]
3S*-(2,4-dihydroxybenzoyl)-4R*,5R*-dimethyl-5-[4-methyl-5-(4-methyl-2-furyl)-3(*E*)-penten-1-yl]tetrahydro-2-furanone	**450**	*F. ferulaeoides*(Steud.) Korov.	[199]
3S*-(2,4-dihydroxybenzoyl)-4R*,5S*-dimethyl-5-[4-methyl-5-(4-methyl-2-furyl)-3(*E*)-penten-1-yl]tetrahydro-2-furanone	**451**	*F. ferulaeoides* (Steud.) Korov.	[199]
8,9-Dihydroxydshamirone	**455**	*F. ferulaeoides* (Steud.) Korov.	[200]
(4*E*,8*E*)-1-(2,4-dihydroxyphenyl)-2-hydroxy-5,9,13-trimethyltetradeca-4,8,12-trien-1-one	**438**	*F. ferulaeoides* (Steud.) Korov.	[198,201]
1-(2,4-dihydroxyphenyl)-3,7,11-trimethyl-3-vinyl-6(*E*),10-do-decadiene-1,9-dione	**440**	*F. ferulaeoides* (Steud.) Korov.	[201]
(6*E*)-1-(2,4-dihydroxyphenyl)-3,7,11-trimethyl-3-vinyl-6,10-dodecadien-1-one	**439**	*F. ferulaeoides* (Steud.) Korov.	[198,201]
(6*E*)-1-(2,4-dihydroxyphenyl)-3,7-dimethyl-3-vinyl-8-(4-methyl-2-furyl)-6-octen-1-one	**441**	*F. ferulaeoides* (Steud.) Korov.	[198,201]
Dshamirone	**437**	*F. ferulaeoides* (Steud.) Korov.	[198,201]
Ferulaeolactone A	**452**	*F. ferulaeoides* (Steud.) Korov.	[200]
Ferulaeone A	**429**	*F. ferulaeoides* (Steud.) Korov.	[198]
Ferulaeone B	**430**	*F. ferulaeoides* (Steud.) Korov.	[198]
Ferulaeone C	**431**	*F. ferulaeoides* (Steud.) Korov.	[198]
Ferulaeone D	**432**	*F. ferulaeoides* (Steud.) Korov.	[198]
Ferulaeone E	**433**	*F. ferulaeoides* (Steud.) Korov.	[198]
Ferulaeone F	**434**	*F. ferulaeoides* (Steud.) Korov.	[198]
Ferulaeone G	**435**	*F. ferulaeoides* (Steud.) Korov.	[198]
		*F. sinkiangensis* K. M. Shen	[21]
Ferulaeone H	**436**	*F. ferulaeoides* (Steud.) Korov.	[198]
Fukanedone A	**424**	*F. fukanensis* K. M. Shen	[18]
Fukanedone B	**425**	*F. fukanensis* K. M. Shen	[18]
		*F. ferulaeoides* (Steud.) Korov.	[198,200]
Fukanedone C	**426**	*F. fukanensis* K. M. Shen	[18]
Fukanedone D	**427**	*F. fukanensis* K. M. Shen	[18]
Fukanedone E	**428**	*F. fukanensis* K. M. Shen	[18]
3-(2-hydroxyl-4-methoxybenzoyl)-4S*,5R*-dimethyl-5-[4,8-dimethyl-3(*E*),7(*E*)-nonadien-1-yl]tetrahydro-2-furanone	**442**	*F. ferulaeoides* (Steud.) Korov.	[198]
8,9-Oxoisopropanyldshamirone	**454**	*F. ferulaeoides* (Steud.) Korov.	[200]
Pallidone C	**444**	*F. pallida* Korovin	[179]
Pallidone D	**445**	*F. pallida* Korovin	[179]
Pallidone E	**446**	*F. pallida* Korovin	[179]
Pallidone F	**447**	*F. pallida* Korovin	[179]
Sinkiangenone A	**448**	*F. sinkiangensis* K. M. Shen	[21]
Sinkiangenone B	**449**	*F. sinkiangensis* K. M. Shen	[21]

**Table 5:** The MIC values of sesquiterpenes and sesquiterpene derivatives against different bacterial strains.

Names	No.	Bacterial Strains	MIC	References
2,3-Dihydro-7-hydroxy-2S*,3R*-dimethyl-2-[4,8-dimethyl-3(*E*),7-nonadienyl]-furo[3,2-*c*]coumarin	**383**	*S. epidermidis*	5.2 μM	[210]
		*M. luteus*	22.5 μM	[210]
(6*E*)-1-(2,4-dihydroxyphenyl)-3,7,11-trimethyl-3-vinyl-6,10-dodecadien-1-one	**439**	*S. epidermidis*	11.2 μM	[210]
		*M. luteus*	22.5 μM	[210]
		*B. subtilis*	11.2 μM	[210]
Diversolide A	**103**	*S. aureus*	>160 µg/mL	[49]
		*E. coli*	80 µg/mL	[49]
Diversolide D	**106**	*S. aureus*	40 µg/mL	[49]
		*E. coli*	>160 µg/mL	[49]
Diversolide F	**108**	*S. aureus*	80 µg/mL	[49]
		*E. coli*	80 µg/mL	[49]
Ethyl galbanate	**215**	*H. pylori*	64 µg/mL	[215]
Fekrynol acetate	**212**	*E. faecium*	128 µg/mL	[215]
Ferulenol	**349**	*M. fortuitum*	2 µg/mL	[207]
		*M. phlei*	2 µg/mL	[207]
		*M. aurum*	2 µg/mL	[207]
		*M. smegmatis*	0.5 µg/mL	[207]
		*B. subtilis*	0.63 µg/mL	[208]
		*S. aureus*	0.63 µg/mL	[208]
		*S. durans*	0.63 µg/mL	[208]
		*S. faecalis*	0.63 µg/mL	[208]
		*Mycobacterium* organisms	1.25 µg/mL	[208]
Ferutinin	**18**	*MRSA*	<0.39 µg/mL	[205]
		*B. subtilis*	<0.39 µg/mL	[205]
		*MTB*	2 µg/mL	[205]
		*BCG*	1.56 µg/mL	[205]
		*M. smegmatis*	10 µg/mL	[206]
Galbanic acid	**213**	*class A β-lactamase*	47 ± 3.1 μM	[214]
4′-Hydroxy kamolonol acetate	**277**	*H. pylori*	64 µg/mL	[158]
		*S. aureus*	64 µg/mL	[132]
Kamolonol	**334**	*H. pylori*	64 µg/mL	[158]
		*S. aureus*	64 µg/mL	[132]
Kamonolol acetate	**278**	*H. pylori*	128 µg/mL	[215]
Methyl galbanate	**214**	*E. faecium*	64 µg/mL	[215]
Teferidin	**17**	*S. aureus*	0.78 µg/mL	[205]
		*B. subtilis*	<0.39 µg/mL	[205]
		*MTB*	0.69 µg/mL	[205]
		*BCG*	3.125 µg/mL	[205]
Teferin	**21**	*MRSA*	1.56 µg/mL	[205]
		*B. subtilis*	1.56 µg/mL	[205]
		*MTB*	8 µg/mL	[205]
		*BCG*	6.25 µg/mL	[205]
Umbelliprenin	**150**	*B. subtilis*	500 µg/mL	[209]
		*B. cereus*	500 µg/mL	[209]
		*E. coli*	500 µg/mL	[209]
		*S. typhi*	500 µg/mL	[209]
		*K. ponumoniae*	500 µg/mL	[209]
		*S. aureus*	500 µg/mL	[209]
		*S. epidermilis*	500 µg/mL	[209]
		*Class A β-lactamase*	54 ± 2.9 μM	[214]

## Data Availability

Not applicable.
